# Reconfigurable Nucleic Acid Nanoparticles with Therapeutic RNAi Responses to Intracellular Disease Markers

**DOI:** 10.1002/adfm.202508122

**Published:** 2025-07-31

**Authors:** Yelixza I. Avila, Anh Ha, Morgan R. Chandler, Nathalia Leal Santos, Taejin Kim, Hannah S. Newton, Marina A. Dobrovolskaia, Kirill A. Afonin

**Affiliations:** Nanoscale Science Program, Department of Chemistry, University of North Carolina at Charlotte, Charlotte, NC 28223, USA; Nanoscale Science Program, Department of Chemistry, University of North Carolina at Charlotte, Charlotte, NC 28223, USA; Nanoscale Science Program, Department of Chemistry, University of North Carolina at Charlotte, Charlotte, NC 28223, USA; MIMETAS US, Inc, Gaithersburg, MD20878, USA; Nanoscale Science Program, Department of Chemistry, University of North Carolina at Charlotte, Charlotte, NC 28223, USA; Center for Translational Research in Oncology (LIM24), Instituto do Câncer do Estado de São Paulo, Hospital das Clínicas da Faculdade de Medicina da Universidade de São Paulo, Comprehensive Center for Precision Oncology, Universidade de São Paulo, São Paulo 01246-000, Brazil; West Virginia University Institute of Technology, Beckley, WV 25801, USA; Nanotechnology Characterization Laboratory, Cancer Research Technology Program, Frederick National Laboratory for Cancer Research, Frederick, MD 21702, USA; Nanotechnology Characterization Laboratory, Cancer Research Technology Program, Frederick National Laboratory for Cancer Research, Frederick, MD 21702, USA; Nanoscale Science Program, Department of Chemistry, University of North Carolina at Charlotte, Charlotte, NC 28223, USA

**Keywords:** KRAS, NANPs, pancreatic cancer, RNAi, survivin

## Abstract

The therapeutic potential of RNA and DNA is evident from numerous formulations approved by the FDA in recent years, with formulations based on RNAi standing out as a successful example. The new class of medicines based on RNAi combines the process of diagnosis and treatment via sequence-specific recognition of biomarker mRNAs and downregulation of their translation. While this approach proved clinically successful, safer, and more personalized options that mitigate adverse effects can be revealed by separating diagnostic and treatment steps. A concept is introduced that allows RNAi therapies to selectively exert their function within diseased cells. The reconfigurable nucleic acid nanoparticles, or recNANPs, recognize overexpressed cancer biomarkers and conditionally release RNAi inducers targeting apoptosis inhibitors in pancreatic cancer cells. RecNANPs are non-immunostimulatory, achieve prolonged gene silencing compared to conventional RNAi inducers, and can be combined with chemotherapy. It is anticipated that this modular platform will enable further advancements in the development of biocompatible nanodevices activated by intracellular variables of choice, facilitating treatment with a repertoire of nucleic acid therapies.

## 1. Introduction

Therapeutic nucleic acids (TNAs) encompass a wide array of functional biomolecules, including antisense oligonucleotides, RNA interference (RNAi) inducers, aptamers, ribozymes, immunomodulatory oligonucleotides, mRNAs, and gene editing tools such as RNA-guided CRISPR/Cas systems.^[1]^ Despite their recent success, TNAs still encounter numerous challenges related to the shortage of suitable technologies for achieving target-specific delivery of precision formulations while minimizing non-specific toxicity and off-target effects.^[2–4]^ For RNAi therapies, such as siRNAs and miRNAs, these limitations primarily arise from their mechanism of action, which involves sequence-specific inhibition of gene expression. All existing RNAi therapeutics integrate a diagnostic step, wherein the RNA-induced silencing complex (RISC) loaded with exogenous RNAs binds to mRNA. Then, the treatment step involves silencing of the function of the same mRNA. Although this approach is practical, with several FDA (U.S. Food and Drug Administration) approvals,^[5–8]^ modular separation of the diagnostic and treatment steps would enhance personalized therapeutic options and improve the safety profiles of TNAs.

Many diseases stem from the dysregulation of gene expression or mutations.^[9–11]^ Differentially expressed genes can serve as signatures to distinguish corrupted cells from healthy tissues. Several design strategies have been developed for nucleic acid nanodevices to recognize specific molecular inputs (e.g., overexpressed mRNAs or miRNAs) and link them to the specific functional outputs.^[12–16]^ However, these approaches rarely leverage the RNAi pathway to elicit a therapeutic response. Previously, we developed and tested RNA logic gates exemplified by two-stranded RNA switches tuned for conditional activation of RNAi in cancer cells.^[17]^ These logic gate switches were engineered to bind to intracellular disease marker mRNAs, initiating conformational changes that lead to the release of shRNA-like constructs, which, upon dicing, produced siRNAs and activated RNAi. While shown to be functional in human cancer cells, the broader use of this approach was limited by an intense computational design, an inability to release multiple siRNAs, and difficulties with scale-up production due to the lengths and sequence limitations of RNA strands. Therefore, a modular, user-friendly platform is needed to establish a cost-efficient strategy adaptable to new biomarkers and multiple targets across various diseases.

**Figure 1.**
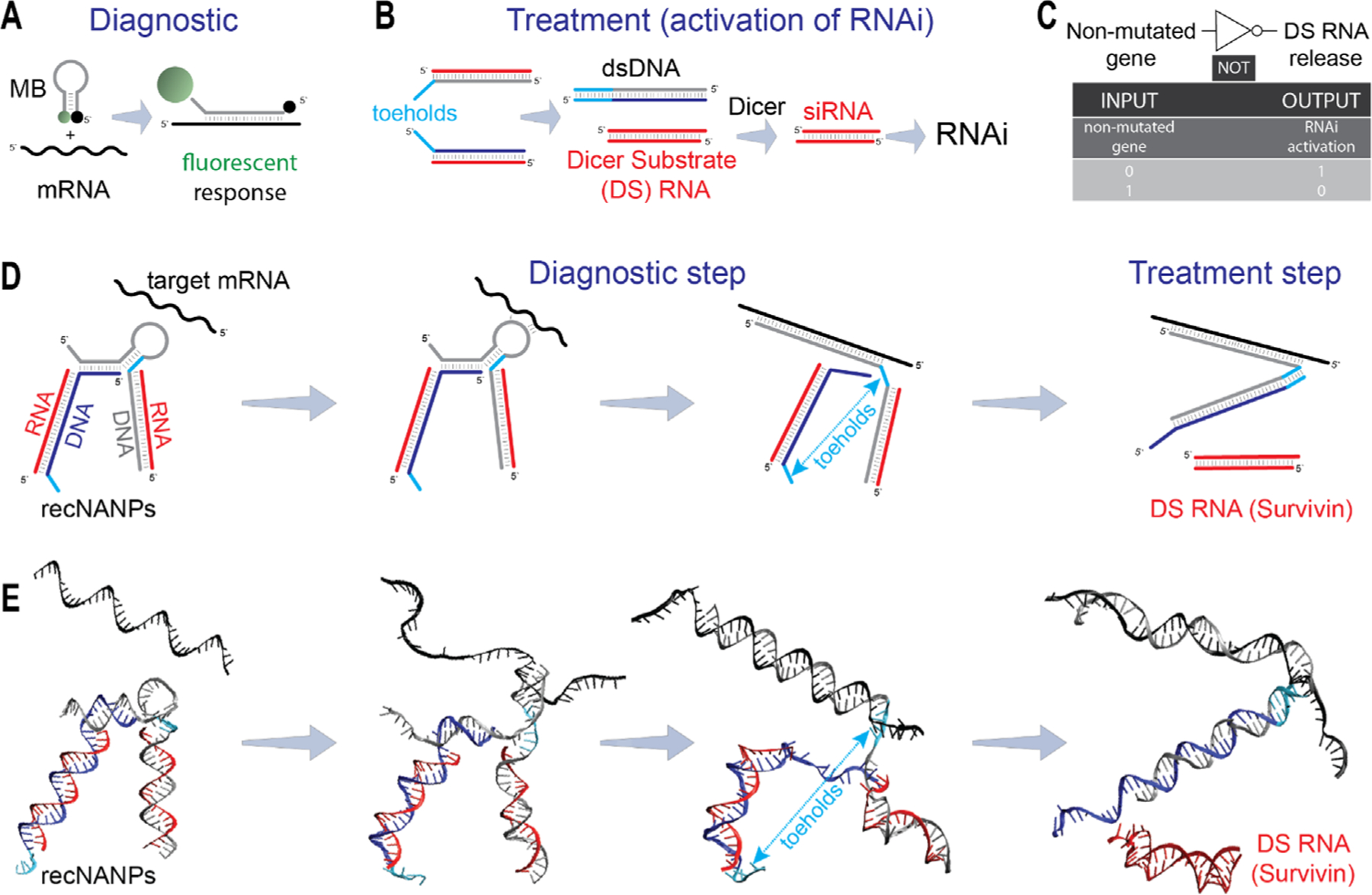
Modular four-stranded reconfigurable nucleic acid nanoparticles (recNANPs) for conditional activation of therapeutic RNAi responses upon intracellular interaction with a target mRNA. (A) Diagnostic – working principle of molecular beacons (MBs). (B) Treatment – working principles of split RNAi inducers. (C) Logic gate rules for input and output products wherein recNANPs act as a simple NOT logic gate. In (D), 2D schematics of recNANPs activation and (E) predicted 3D structures are shown for all constructs.

A prominent demonstration of conditional reporting of diseased biomarkers is molecular beacons (MBs).^[18–22]^ These probes (Figure 1A), crafted from short DNA oligos and their chemical analogs, possess the unique ability to detect and signal the presence of specific nucleic acid sequences that are the subject of investigation. MBs are designed in stem-loop structures with embedded fluorophore/quencher pairs that are separated once the MBs bind the target. This leads to heightened fluorescence, effectively reporting the disease. MBs play an essential role in medical diagnostics. This well-established technology has led to the development of hundreds of MBs targeting a range of biomarkers, both intracellular and extracellular. Thus, the versatility and specificity of validated MBs make them ideal candidates for incorporation into a diagnostic module of novel reconfigurable devices.

Building upon split-protein technology,^[23–25]^ we introduced a novel concept^[26]^ that enabled the conditional activation of multiple functionalities within nucleic acid nanoparticles (NANPs).^[26–37]^ This innovation relies on RNA/DNA hybrids that communicate with each other through sequence complementarity, triggering the activation of different functionalities both in vitro and in vivo.^[26]^ The core idea involves splitting functional entities such as RNAi inducers, Förster resonance energy transfer (FRET) pairs of dyes, and aptamers into inactive RNA/DNA hybrids. These hybrids, equipped with complementary single-stranded toeholds, initiate re-association upon encountering their cognate partners, restoring the intended function via isothermal strand displacement (Figure 1B).^[26,38–42]^ Several teams have adapted this concept and applied it in their research.^[35,36,43]^ In this current work, we implement the split RNAi inducers as a treatment module for our nucleic acid constructs.

Despite the existing assortment of therapeutic RNA and DNA nanoparticles and computational tools available for their design,^[17,44–50]^ it is important to note that most of them remain static in nature. They lack the capability to dynamically interact with biological systems and conditionally respond to intracellular environments. We now introduce a system that amalgamates two well-established technologies within a single nanostructure: MBs for diagnostic and split RNAi for treatment. The resulting reconfigurable NANPs, or recNANPs, can dynamically respond to disease-associated cellular environments and activate targeted TNAs through NOT logic (Figure 1C). The NOT gate implemented into the current recNANPs follows the truth table in which the release of TNAs is true only if the non-mutated gene is false. Thus, in the absence of a disease-associated trigger, the recNANPs remain inactive. However, recNANPs are equipped with sequence regions that can bind to the overexpressed intracellular oncogene (*KRAS* G12D), inducing conformational changes that lead to the release of siRNAs against apoptosis inhibitor genes (Figure 1D; Movies S1 and S2, Supporting Information). The G12D *KRAS* mutation is found in up to 35% of patients with pancreatic cancer,^[51]^ making it a valuable diagnostic marker tested in this work.

We demonstrate the design, production, and optimization of storage conditions for recNANPs. We then validate predicted interactions with target strands, and recNANPs’ ability to intracellularly recognize overexpressed prognostic cancer triggers to release RNAi inducers targeting apoptosis inhibitors in both conventional 2D cell cultures and more advanced 3D spheroid models. We assess the duration of the recNANPs therapeutic effect, compatibility with chemotherapeutics, and immunorecognition by freshly isolated human peripheral blood mononuclear cells (PBMCs). Our work highlights several innovative aspects of this technology and underscores the adaptability of recNANPs’ design, enabling the simple replacement of therapeutic domains to address evolving therapeutic needs. Our findings contribute to advancing the field of RNAi therapies and nucleic acid technologies by offering a versatile and targeted approach for treating cancer and other diseases.

## 2. Results and Discussion

### 2.1. Design and Concept Validation of recNANPs

For the diagnostic step, we selected MBs designed to exclusively recognize endogenous *KRAS* codon 12 mutation (GGT→GAT, G12D).^[51–56]^ The function of the chosen MBs was validated in cells for the specific detection of pancreatic cancer.^[57,58]^ For the treatment step, we have designed Dicer Substrate (DS) RNAs,^[59]^ using our established protocols,^[33]^ that upon intracellular dicing, release siRNAs targeting Survivin. Survivin is a potent inhibitor of apoptosis reported in 77% of pancreatic ductal adenocarcinomas.^[60–62]^

**Figure 2.**
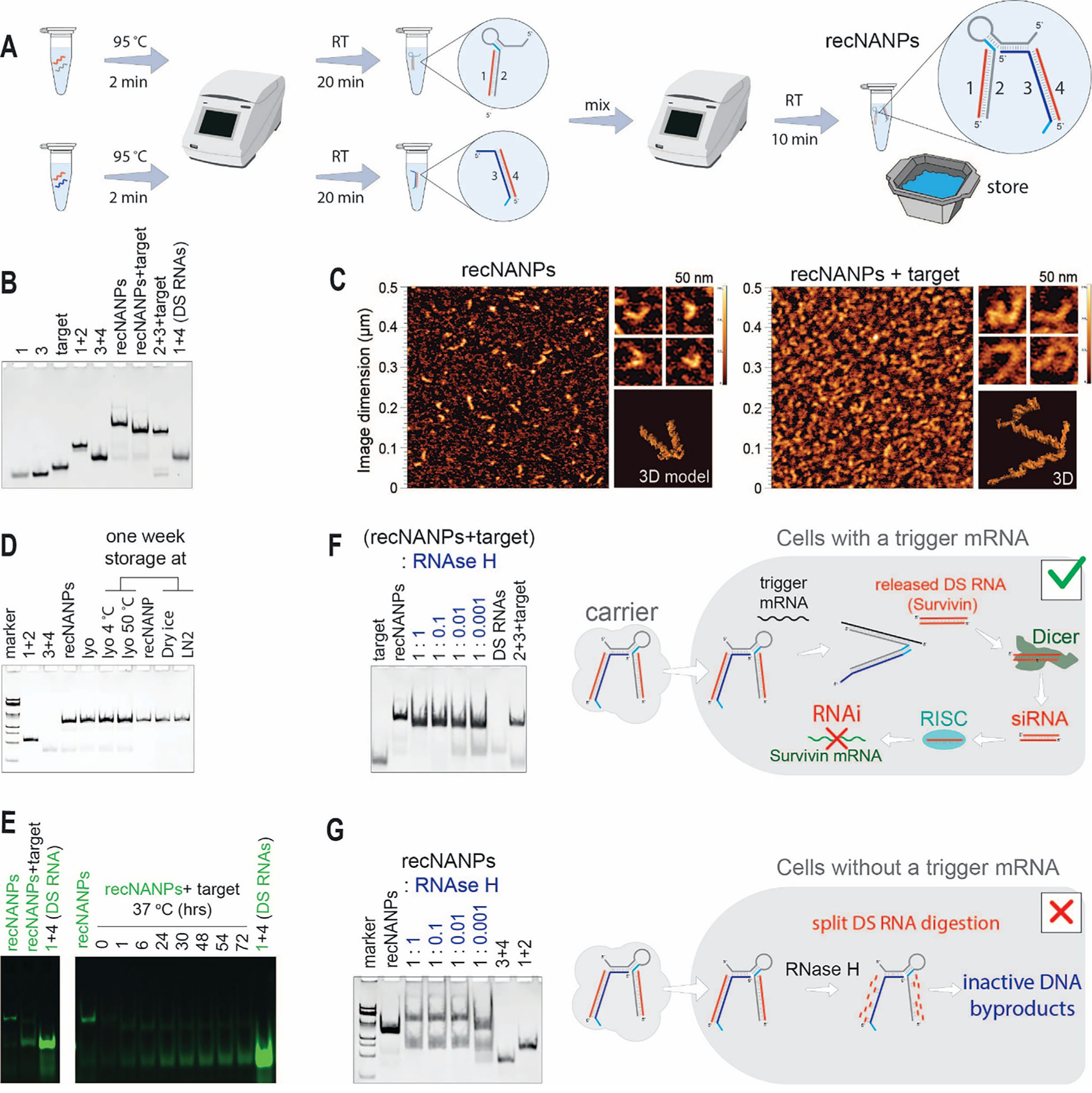
Assembly and in vitro characterization of recNANPs. (A) Schematic depicting the recNANPs’ assembly protocol. (B) EtBr-stained native-PAGE confirming assembly of recNANPs and conditional release of DS RNAs. (C) AFM of recNANPs (left) and recNANPs incubated with target sequence (right), showing the morphological changes between inactive and activated states. (D) The stability of recNANPs was monitored over one week under various storage conditions, including lyophilization (lyo) and storage in liquid nitrogen (LN2). (E) Activation of recNANPs after introducing the target sequence is shown for Alexa 488-labeled recNANPs and DSRNAs. (F) Competition between triggered release of DS RNAs and digestion of recNANPs by RNAse H. (G) Digestion of recNANPs with RNAse H in the absence of the target strand. The higher-order bands may correspond to larger assemblies of inactive DNA byproducts, as suggested by the predicted models shown in Figure S3 (Supporting Information).

We have engineered and tested recNANPs assembled from four short, chemically synthesized oligos (Figure 2A), where DNA strand 2 incorporates a stem-loop region of MB complementary to G12D KRAS^[57,58]^ and another region complementary to the DS RNA sense (strand 1) sequence. This strand 2 is also elongated at the 5′-end to facilitate binding to DNA strand 3, which carries the complement for split DS RNA antisense (Strand 4). The interactions between complementary DNA toeholds, highlighted in light blue (Figure 1D), which are protected by the stem of the inactive MB, are necessary for RNA-DNA hybrid reassociation and DS RNA release after the diagnostic step is achieved. These toeholds become mutually accessible only upon complete binding and opening of the stem loop to the target mRNA, facilitating the release of DS RNA (duplex 1–4) through isothermal reassociation of separated RNA/DNA duplexes 1–2 and 3–4 (Figure 1D).

To optimize the assembly conditions of recNANPs, we compared various annealing protocols, confirming the results through electrophoretic mobility shift assays using native-PAGE. The stepwise assembly process (Figure 2A,B) started with the formation of duplexes 1+2 and 3+4, followed by their incubation at different temperatures (Figure S1, Supporting Information). Notably, recNANPs efficiently assembled both at lower (e.g., 4 °C) and higher (e.g., 45 °C) temperatures when incubated immediately after duplex combination. Since no significant differences in assembly yields were observed, incubation at room temperature (RT) was selected as the optimal assembly condition.

Native-PAGE and AFM were used to analyze recNANP structures before and after interaction with the target strand (Figure 2B,C,E). Both techniques revealed uniform morphology of recNANPs, which changed upon target introduction, producing the re-associated and predicted structures, including therapeutic DS RNAs and 2+3+target byproducts (corresponding representative 3D models shown).

Cold chain storage is essential for maintaining the potency of conventional TNAs and NANPs, yet it significantly increases transportation and handling costs. Therefore, we optimized the preparation of recNANPs for extended handling at ambient temperatures. Previous studies have identified optimal dehydration protocols for various NANPs, aiding in maintaining their structural stability at higher temperatures over extended periods of time.^[63–65]^ Here, we tested different dehydration protocols, including speed-vac drying and lyophilization, and compared them to recNANPs stored in solutions. All samples were stored across a broad temperature range (−80 to 50 °C), and their integrity was assessed (Figure 2D; Figure S2, Supporting Information). The results indicate that lyophilized recNANPs and frozen recNANPs are the optimal storage condition options to maintain function over time.

Given that intracellularly delivered recNANPs will compete with cytosolic nucleases before and during their activation by target strands, we explored the effects of enzymatic degradation versus the release of DS RNAs from recNANPs. We tested RNAse H, an enzyme known to hydrolyze RNA strands within RNA/DNA hybrids (Figure 2F) to assess whether recNANP activation could outpace enzymatic degradation. Additionally, we evaluated the effect of RNase H on recNANPs in the absence of target strands to rule out off-target DS RNA release (Figure 2G). The recNANPs demonstrated DS RNA release only in the presence of target strands (Figure 2F), and no DS RNA was detected when recNANPs were exposed to RNase H without target strands added (Figure 2G).

### 2.2. Cellular Uptake, Conditional Activation, and Silencing Efficiency

**Figure 3.**
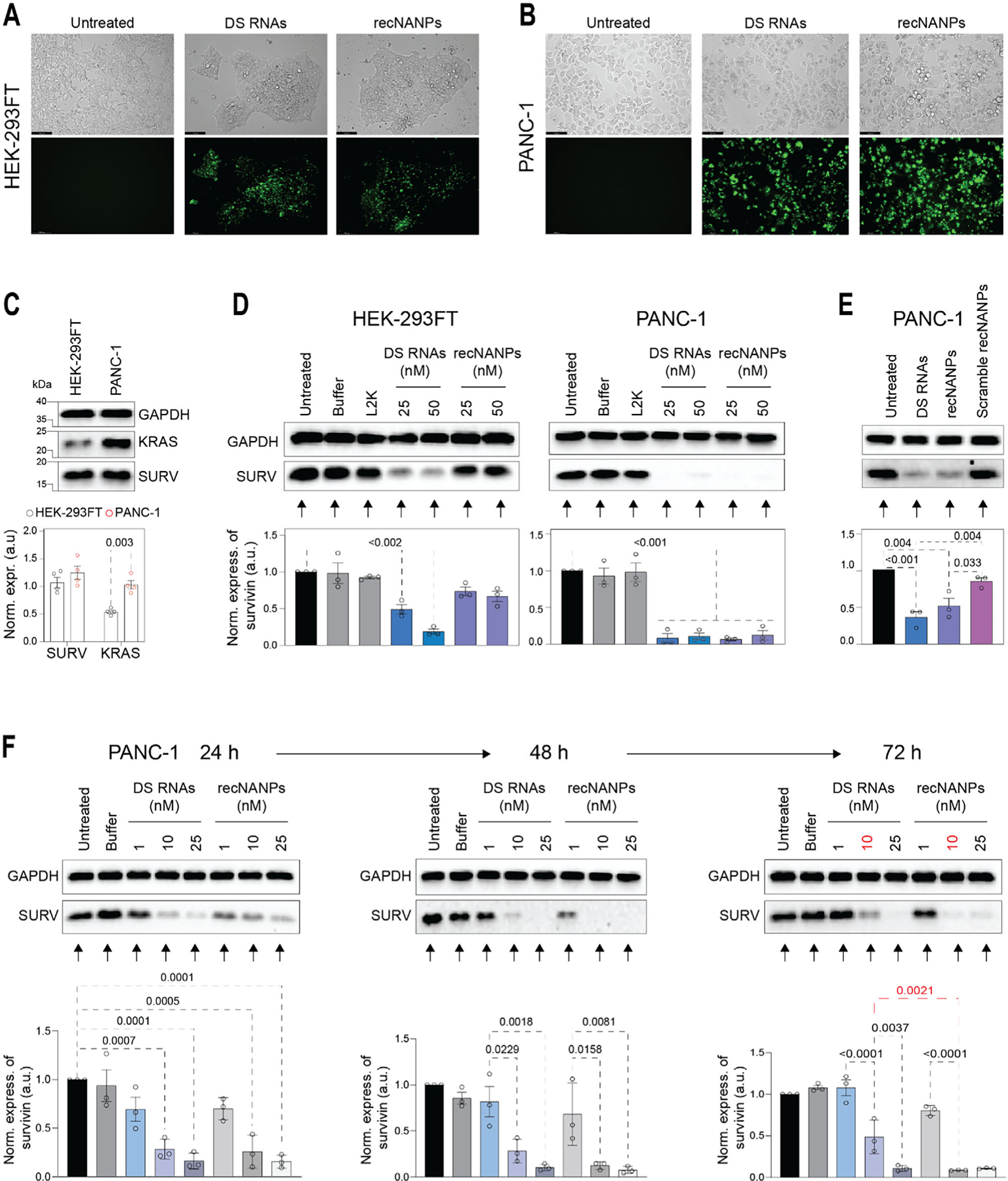
Cellular uptake and specificity of activation of recNANPs. (A-B) Microscopy of uptake in HEK-293FT and PANC-1 cells of Alexa 488-labeled DS RNAs and recNANPs (green) after 72 h of incubation at 25 nM. (C) Relative expression of Survivin and KRAS in HEK-293FT and PANC-1 cells. (D) Expression of Survivin in HEK-293FT and PANC-1 cells after treatment with DS RNA and recNANPs, with media replaced after 24 h for a total of 72 h incubation, was analyzed by western blots. (E) Survivin expression in PANC-1 cells 24 h after treatment with DS RNAs, recNANPs, or recNANPs containing scrambled stem-loop regions (all at 25 nM). Representative immunoblots demonstrate changes in the expression of Survivin at the predicted size of 16.5 kDa (Figure S6, Supporting Information). Each bar represents the mean of N = 3 biological replicates ± SEM. Statistical significance was assessed using one-way ANOVA and is indicated by numerical p-values. (F) Expression of Survivin in PANC-1 cells at 24, 48, and 72 h. Each bar is the mean of N = 3 biological repeats ± SEM (Figures S8–S10, Supporting Information). Statistical significance is shown (t-test).

Two different cell lines were selected to study the sequence-specific activation of recNANPs. PANC-1 cells, which carry the G12D *KRAS* mutation, were chosen as our target, and HEK-293FT cells were selected as the wild-type (WT) KRAS control cell line.^[51–56]^ We first compared the relative transfection efficiencies of recNANPs and DS RNAs labeled with Alexa 488 using fluorescence microscopy (Figure 3A,B) and flow cytometry (Figures S4 and S5, Supporting Information). Flow cytometry analysis of PANC-1 cells revealed that DS RNAs achieved a higher transfection efficiency (≈90%), while recNANPs exhibited reduced uptake efficiency (≈65 to 70%) for the same concentrations. In addition, the percentage of cells that died due to uptake from DS RNAs was higher than recNANPs (≈7% vs ≈4%), corresponding to the transfection efficiency observed. In HEK-293FT cells, DS RNAs also displayed significantly higher uptake at 48 h than recNANPs (≈66% vs ≈30%), with cells dying due to uptake of recNANPs at less than 1%.

To understand how target abundance influences recNANP activation, we first compared the basal expression levels of Survivin and KRAS in HEK-293FT and PANC-1 cells. While Survivin levels were comparable between the two lines (Figure 3C), KRAS expression was significantly lower in HEK-293FT cells. This difference introduced a new variable for evaluating recNANP activation, as low KRAS levels may limit the availability of target strands needed to trigger DS RNA release. To explore this further, we assessed KRAS and Survivin expression in HEK-293FT, PANC-1, and HeLa cells (Figure S6A, Supporting Information), and found that HeLa and PANC-1 cells exhibited similar KRAS expression. We then tested an alternative construct, recNANP-WT, designed to respond to wild-type KRAS, and examined its activity in HeLa cells. Treatment with either recNANP or recNANP-WT resulted in Survivin downregulation (Figures S6B and S7, Supporting Information), indicating that both constructs successfully released DS RNAs. These findings suggest that KRAS expression level is a critical determinant of recNANP activation but also reveal that the current design lacks sufficient sequence discrimination between mutant and wild-type KRAS.

To investigate the relationship of KRAS levels and recNANPs activation, HEK-293FT and PANC-1 cells were treated for 72 h at concentrations of 25 and 50 nM to determine the effect of recNANPs on the regulation of Survivin. In HEK-293FT cells with low KRAS expression, only the DS RNAs exhibited a notable effect on Survivin silencing, while recNANPs remained inactive (Figure 3D; Figure S9, Supporting Information), showing no statistically significant (P>0.05) downregulation of Survivin expression. However, in PANC-1 cells with a high KRAS level, the expression of Survivin was significantly decreased when treated with both DS RNAs and recNANPs, indicating that recNANPs are equally effective as DS RNAs in downregulating Survivin through the RNAi pathway in cells with overexpressed KRAS. This is an addition confirmation that the level of KRAS expression determines the activation of recNANPs.

To confirm that activation is driven by sequence complementarity, we designed a scrambled version of the stem-loop region and incubated these scrambled recNANPs with either KRAS target strands or mock sequences (Figure S8, Supporting Information). As noted above, while recNANPs show limited sequence discrimination for point mutations, no DS RNAs release was observed from the scrambled recNANPs under any condition. Moreover, no changes in scramble recNANPs migration were detected upon addition of target strands, and the treatment of PANC-1 cells with scrambled recNANPs did not result in significant (P = 0.4065) downregulation of Survivin (Figure 3E), further supporting that recNANPs reconfiguration depends on specific hybridization between the loop region and the target strand.

A time-course experiment transfecting PANC-1 cells with DS RNAs and recNANPs (1, 10, and 25 nM) over 24, 48, and 72 h shows significant Survivin downregulation starting at 10 nM by 24 h. At 25 nM, both constructs strongly reduced Survivin (Figure 3F; Figure S10, Supporting Information). By 48 h, recNANPs at 10 nM significantly outperformed DS RNAs, with both achieving complete downregulation at 25 nM. At 72 h, DS RNA effects waned, with Survivin expression returning at 10 nM, while recNANPs maintained suppression. This suggests that recNANPs provide more sustained gene silencing, crucial for cancer therapies. Notably, DS RNAs at 25 nM matched recNANPs at 10 nM, emphasizing recNANPs’ superior efficacy.

### 2.3. Uptake and Conditional Gene Silencing in 3D Spheroids

**Figure 4.**
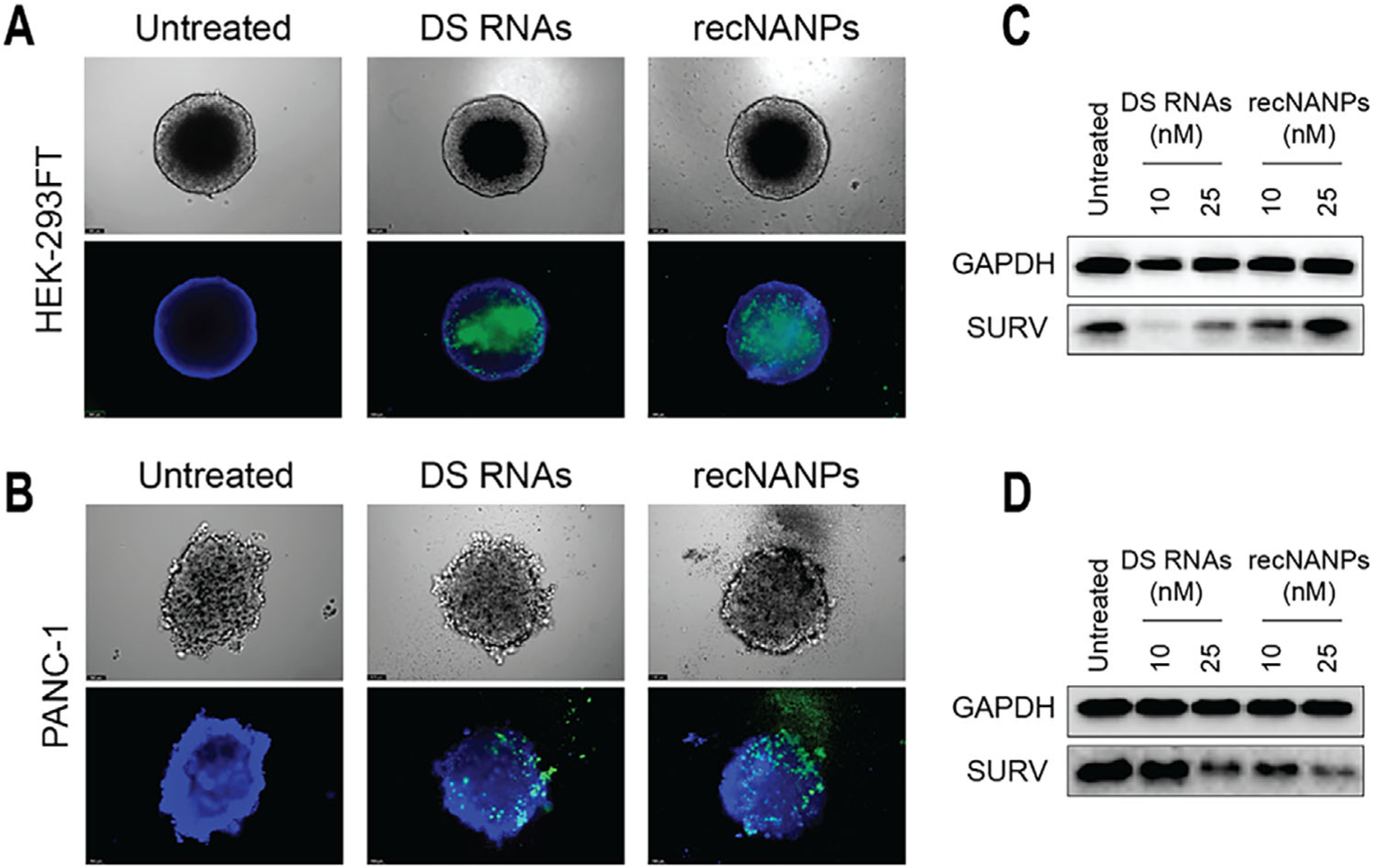
Assessment of uptake and gene silencing in spheroids. Microscopy images of HEK-293FT (A) and PANC-1 (B) spheroids treated with Alexa-488 labeled DS RNAs and recNANPs (green) after 24 h of incubation. The spheroids were washed and stained with NucBlue (blue) prior to imaging. Western blot analysis of treated with DS RNAs and recNANPs HEK-293FT spheroids at 48 h (C) and PANC-1 spheroids at 72 h (D).

The efficient accumulation and penetration of nanoparticles into targeted tissues are crucial for their effective application in clinical settings. Compared to traditional 2D monolayers, 3D cell culture models, such as spheroids, provide a more physiologically relevant environment, better replicating biological barriers and accurately reflecting the challenges in achieving therapeutic efficacy.^[66–68]^ To assess the effect of DS RNAs and recNANPs in a 3D setting, HEK-293FT and PANC-1 spheroids were generated using agarose-coated 96-well plates. Transfection efficiency was evaluated by fluorescence microscopy at 24- and 48-h post-transfection. Before washing, fluorescence imaging revealed Alexa-488 positive cells surrounding the spheroids, particularly for PANC-1 treated with recNANPs, suggesting a higher cytotoxic effect in these cells compared to HEK-293FT (Figure S11, Supporting Information). To confirm intracellular uptake and rule out surface-bound lipoplexes, spheroids were washed with PBS and stained with NucBlue. Following an additional wash, fluorescence microscopy confirmed the uptake of both DS RNAs and recNANPs (Figure 4A,B). Western blot analysis was then performed to assess Survivin downregulation in spheroids. Consistent with monolayer results, the highest concentration of recNANPs did not induce a significant reduction in Survivin expression in HEK-293FT spheroids (Figure 4C), while in PANC-1 spheroids, recNANPs effectively downregulated Survivin (Figure 4D).

### 2.4. Combinatorial Treatment of recNANPs with Chemotherapeutics

**Figure 5.**
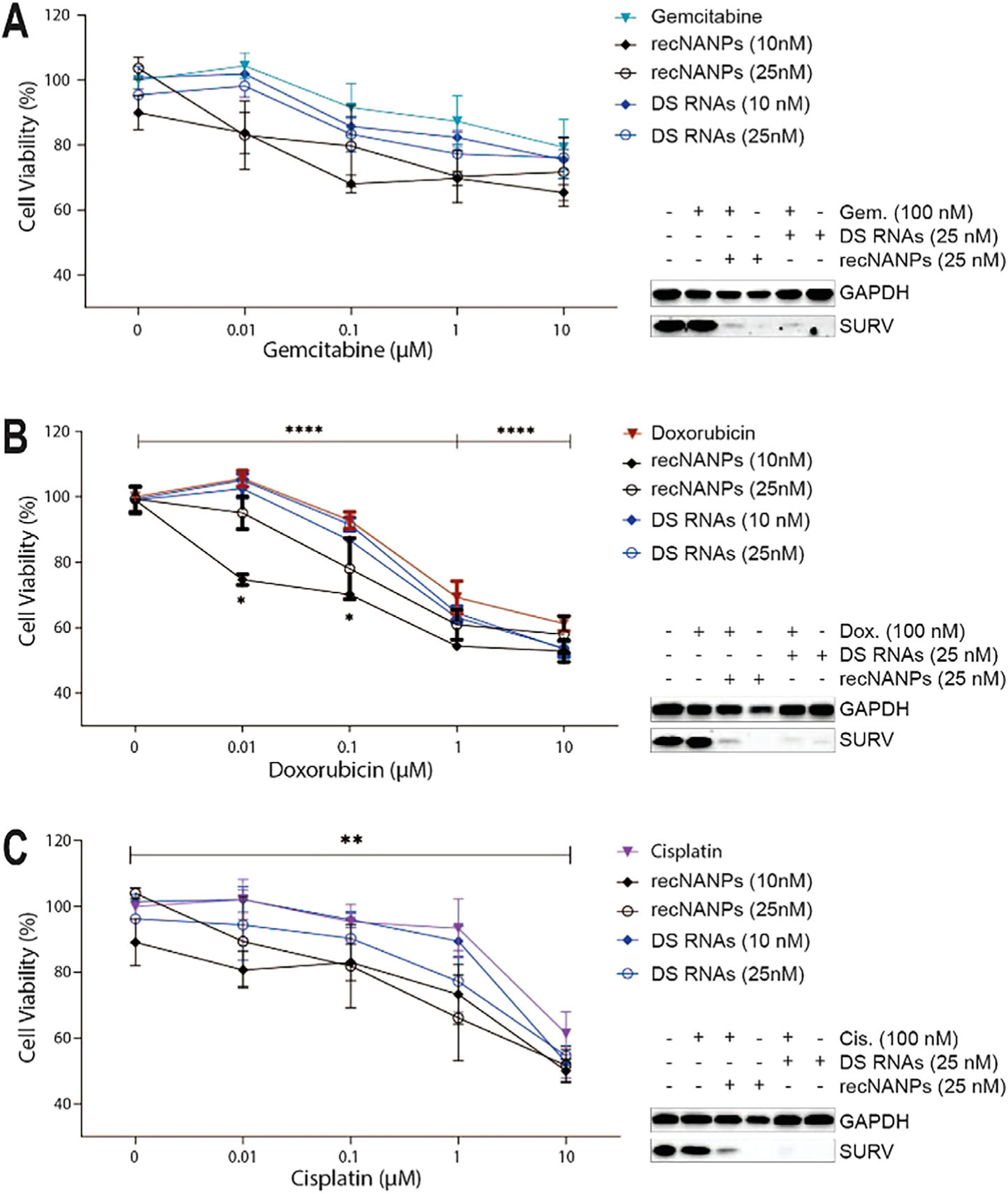
Combinatorial treatment of PANC-1 cells with chemotherapeutics and recNANPs. Cell viability and resulting expression of Survivin of PANC-1 cells 72 h after being treated with (A) gemcitabine (B) doxorubicin, and (C) cisplatin at increasing concentrations (each data point represents the mean of N = 3 biological repeats ± SEM). Each chemotherapeutic was combined with either recNANPs at 10 or 25 nM, or DS RNAs at 10 or 25 nM. Ordinary one-way ANOVAs were conducted to assess statistical significance using GraphPad Prism. P-values were considered as follows: **** P < 0.0001, ** P < 0.004, * P < 0.02.

The therapeutic potential of recNANPs was evaluated by combining them with chemotherapeutics (gemcitabine, doxorubicin, and cisplatin) to assess cell viability effects. Gemcitabine, the gold standard first-line treatment for pancreatic cancer, is often used in combination with other drugs.^[69,70]^ While effective, these drugs have systemic toxicity. To avoid such side effects, we attempted to combine these chemotherapeutic drugs with recNANPs to enhance cell killing at much lower doses. First, PANC-1 cells were treated with various drug doses or DSRNA/recNANPs to determine the range of concentrations at which the cell viability was not much affected (Figures S12 and S13, Supporting Information). Following this, chemotherapeutics were individually supplemented with either DS RNAs or recNANPs (at 10 and 25 nM) for 72 h (Figure 5). These combinations significantly reduced cell viability compared to chemotherapeutics alone. Among the treatments, gemcitabine had the least effect on PANC-1 cell viability, indicating resistance. In contrast, doxorubicin was the most effective, reducing viability by 50% in both cell lines after 72 h, with cisplatin showing a comparable reduction. Combining 10 nM recNANP with 0.01 μM Doxorubicin or Cisplatin further significantly enhanced cell killing, suggesting an additive effect. Western blot analysis confirmed that chemotherapeutic-nucleic acid combinations led to efficient Survivin downregulation, which was not observed during the treatment with the drug alone.

### 2.5. Immune Recognition of recNANPs

**Figure 6.**
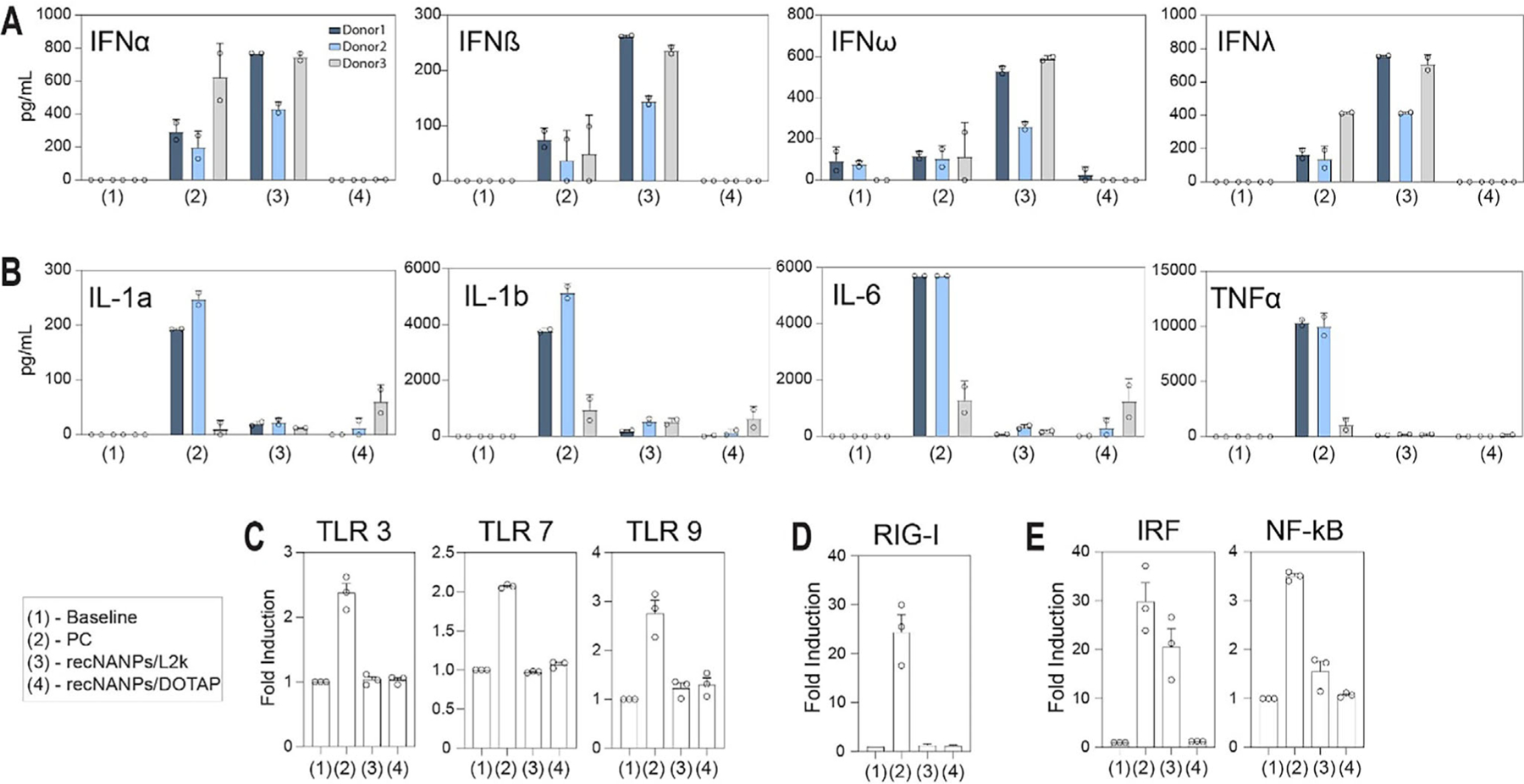
Delivery vehicle impacts immune recognition. PBMCs from healthy human donors were treated with negative control (NC), positive control (PC), and recNANPs delivered with either Lipofectamine (L2K) or DOTAP for 24 h. Supernatants were then analyzed for the presence of cytokines, chemokines, and interferons using multiplex ELISA. The data are presented based on their function, such as (A-B) type I and type III interferons, where each bar shows the mean response and SD (N = 2). Human reporter cell lines were used to assess (C) TLR activation, (D) RIG-I activation, and (E) IRF and NF-*κ*B activation. Each bar shows the mean response ± SEM (N = 3).

Furthermore, in line with recent studies indicating the immune stimulation potential of NANPs designed for therapeutic purposes,^[71–73]^ we conducted experiments to assess the effects of our recNANPs on human PBMCs for the production of various cytokines. We specifically chose this experimental model because PBMCs confer a higher degree of accuracy when predicting cytokine storm toxicity compared to common preclinical animal models like rodents and primates.^[74–76]^ Additionally, we investigated how the use of different carriers might influence the immune recognition of NANPs, as noted in previous works.^[77,78]^ Using another lipid-based delivery agent, DOTAP-liposomes (Figure S14, Supporting Information), we demonstrate that the spectrum of cytokines induced by the same NANPs but delivered using different carriers influences the immune response to the recNANPs. Particularly, recNANPs delivered using L2K induced robust type I and type III interferon response, which included IFN*α*, IFN*β*, IFN*λ*, and IFN*ω*, whereas DOTAP-liposome complexed recNANPs did not have this property (Figure 6A). These data agree with our earlier study comparing L2K-delivered NANPs to their counterparts delivered using amine-terminated PAMAM dendrimers.^[77]^ However, in contrast to the previous study in which amine-terminated PAMAM dendrimer delivered NANPs induced strong pro-inflammatory responses consisting of danger signals and pyrogenic cytokines such as IL-1*α*, IL-1*β*, TNF, IL-6,^[77]^ recNANPs delivered using DOTAP liposomes did not induce substantially high levels of IL-1*α*, IL-1*β*, TNF, or IL-6 (Figure 6B). Since L2K, amine-terminated PAMAM dendrimers, and DOTAP liposomes contain cationic moieties, these data suggest that the zeta potential and the presence of cationic moieties per se are not the main factors driving such differences in responses. Rather, particle composition – lipid-based (e.g., DOTAP liposome) vs polymer-based (PAMAM dendrimer) versus lipid-polymer hybrid L2K – and architecture – spherical (e.g., DOTAP liposomes) vs branched, organized into a well-defined shape (e.g., PAMAM dendrimer) versus complex hybrid material (e.g., L2K) – contribute to the difference in the cytokine responses. Collectively, these data suggest that, in addition to the NANP physicochemical properties and fine differences between lipofectamine carriers, other compositional and architectural variations in the NANP delivery carriers provide additional tools for fine-tuning application-specific, desired cytokine responses and reducing undesirable cytokines for a given application.

Mechanistic studies using reporter cell lines targeting the most common nucleic acid pattern recognition receptors revealed no detection of recNANPs by endosomal TLRs 3, 7, and 9 (Figure 6C). Evaluation of the cytosolic RIG-I receptor similarly showed no detectable activation (Figure 6D). However, as illustrated in Figure 6E, activation of the IRF pathway exhibited comparable trends to those observed in PBMCs, indicating that L2K utilization enhances immune activity over DOTAP. These findings underscore the critical role of carrier selection, suggesting that optimizing carrier choice could bolster the therapeutic effectiveness of recNANPs. To ascertain that the lack of activation was not attributable to diminished cell viability, MTS assays were conducted (Figure S15, Supporting Information), revealing no significant impact on cell viability. The inactive recNANPs comprise DNA/RNA hybrid structures that evade detection by conventional nucleic acid sensors.

THP1-Dual cells, a leukocyte monocytic cell line used to investigate activation of the NF-*κ*B and IRF pathways, were utilized to reveal more information about the immune stimulation pathways of the recNANPs. The cells also carry an NRAS G12D mutation, which is found in the conserved region where the G12D mutation occurs in KRAS in PANC-1 cells.^[79–81]^ As such, we hypothesized that our recNANPs would activate and reconfigure in this reporter cell line to release their DS RNAs. Our results indicate that such reconfiguration is taking place (Figure 6E) based on the activation of the IRF pathway.

### 2.6. Modularity of recNANPs Technology

To further explore the modularity of the recNANPs, we altered their therapeutic module to target BCL-2, another protein linked to the diseased state of pancreatic cancer cells.^[82–84]^ We maintained the same experimental conditions used in the previous silencing experiments. The results demonstrated that the recNANPs maintained specificity for PANC-1 cells, downregulating BCL-2 (Figure S16, Supporting Information).

## 3. Conclusion

In this study, we demonstrated the functionality of a novel class of reconfigurable, smart nucleic acid nanomaterials that respond to intracellular cues, such as differentially expressed genes, by altering both their overall properties and local behavior within human cells. As a proof of concept, we employed a stem-loop region of MBs designed to trigger recNANP reconfiguration and the release of therapeutic DS RNAs in response to KRAS overexpression. Additionally, we combined recNANPs with a standard-of-care cancer chemotherapy drug, doxorubicin to enhance the therapeutic efficacy of both compounds while minimizing systemic toxicity, and demonstrated that the combination significantly impacts cell viability of cancer cells in the PANC-1 model. These results align with earlier findings in the literature, suggesting that integrating functionalized nanomaterials with conventional therapies can potentiate therapeutic outcomes by leveraging both the inherent potency of the chemotherapeutic agents and the versatile properties of nanomaterials.^[85–89]^

We also explored the immune response of recNANPs in PBMCs and TLR cell lines using two different delivery vehicles: DOTAP and L2K. It was observed that the delivery vehicle affects immune recognition in both immune response models. DOTAP, a cationic lipid used for transfections, resulted in lower levels of immune activation compared to when L2K was used as a carrier. This result is consistent with previous studies that have highlighted the importance of delivery vehicles in modulating immune responses to nucleic acid-based therapies.^[72,77,78]^

Taken together, these studies suggest that the recNANP platform offers a strategy for decoupling the diagnostic and therapeutic functions of gene silencing, positioning it as a versatile approach for RNAi therapeutics. Although the current design depends on elevated KRAS levels for activation, it does not sufficiently discriminate between mutant sequences. We attribute this outcome to the minimal thermodynamic difference between mutant and wild-type KRAS binding to the stem-loop region of the recNANPs, combined with a balance between recNANP activation and enzymatic degradation. This underscores the critical roles of target abundance and cellular processing capacity. These findings highlight the need for further refinement of recNANP design to enhance sequence selectivity while preserving structural integrity. We anticipate that recNANPs will have far-reaching implications across a range of biomedical applications, including cancer therapies. Beyond medicine, their customizable nature positions them for use in synthetic biology, tissue engineering, and therapeutic interventions, including cell fate modulation. Future efforts will focus on optimizing the stem-loop region and overall recNANP architecture to improve specificity, increase solution stability, and achieve precise control over target-specific DS RNA release. Incorporation of chemical modifications represents a promising strategy toward these goals. The modularity of the recNANP platform facilitates adaptation to target a wider range of disease-associated proteins and biomarkers implicated in cancer and inflammatory diseases, thereby broadening its potential applications. Through these advancements, recNANPs could evolve into a versatile and robust tool for precise molecular modulation across diverse disease models.

## 4. Experimental Section

### recNANP Synthesis, Verification, and Stability Testing:

All oligos, as listed in the supplementary information, were purchased from Integrated DNA Technologies, Inc. Each strand was purified through denaturing polyacrylamide gel electrophoresis (PAGE, 8%) in the presence of 8 M urea, 89 mM tris-borate, and 2 mM EDTA. Bands were visualized using UV shadowing, excised, and eluted into a solution containing 300 mM NaCl, 89 mM tris-borate, and 2 mM EDTA overnight at 4 °C. The following day, samples were precipitated by adding two volumes of 100% ethanol, chilling to −20 °C for a minimum of 3 h, spinning at 14 000 g for 30 min, and discarding the supernatant. An additional washing step was performed with 90% ethanol, followed by centrifugation and supernatant disposal. The samples were then processed using SpeedVac to remove any remaining ethanol. Finally, the oligo strands were redispersed in endotoxin-free water, and their concentrations were measured using Nanodrop 2000.

The assembly of four-stranded recNANPs occurs in two steps. In the first step, intermediate RNA/DNA duplexes composed of strands 1+2 and 3+4 were assembled separately. For each intermediate, corresponding monomers were combined in equimolar ratios in assembly buffer (89 mM tris-borate, 2 mM MgCl_2_, 50 mM KCl), heated to 95 °C for 2 min, and then incubated at room temperature for 20 min. Next, the 1+2 and 3+4 duplexes were combined in equimolar ratios and incubated at room temperature (RT) for 10 min (Figure 2A).

To confirm the assembly of all structures, all samples were run in a non-denaturing native-PAGE (8%, 37.5:1) in 89 mM tris-borate (pH 8.2), and 2 mM MgCl_2_ at 4 °C. After electrophoresis, the gel was stained with ethidium bromide and visualized using a ChemiDoc MP Imaging System.

Once recNANPs were assembled, a mock target sequence monomer was introduced at an equimolar ratio to induce the conformational change and release of the DS RNAs during the incubation at 37 °C. The aliquots were taken at different timepoints, snap frozen on dry ice, and then loaded in reverse order and run on a native-PAGE to investigate at what point the DS RNA was released from recNANPs.

To assess the effect of a scrambled stem-loop sequence on recNANP activation, Alexa 488-labeled scramble recNANPs were incubated with either target strands or mock strands at 37 °C for 15–16 h. Samples were then analyzed following the protocol described above.

Atomic force microscopy (AFM) was employed to examine the morphology of the recNANPs. Mica substrates were freshly cleaved and treated with 1-(2-aminopropyl) silatrane (APS) following established protocols to ensure proper surface preparation. AFM imaging was then performed using the procedures outlined in previous publications.^[90,91]^

### recNANP Target Versus Enzymatic Activation:

recNANPs were incubated with different dilutions (1:1, 1:10, 1:100, 1:1000) of RNase H (NEB#M0297, 5000units mL^−1^) for 24 h at 37 °C, followed by loading into 8% non-denaturing PAGE (37.5:1, 89 mM tris-borate (pH 8.2), 2 mM MgCl_2_). Samples were run at 180 V for 45 min at 4 °C using the Mini-PROTEAN Tetra system (Bio-Rad, Hercules, CA, USA). Gels were stained with ethidium bromide for 5 min to visualize using a ChemiDoc MP system. A similar protocol was repeated for recNANPs with mock target at 37 °C for 24 h with RNase H under the same dilutions listed.

### Molecular Dynamic Simulations:

Discovery Studio Visualizer built the initial structures of each step of the recNANPs and their activation. Two Amber force fields, DNA.OL15^[92]^ and RNA.OL3,^[93]^ were used to simulate DNA and RNA strands, respectively. Energy minimization was applied to repair atomic crashes in the initial structures of each step. In each TMD simulation, two nucleotides in a base pair in the starting conformation were dissociated and were re-paired with target nucleotides. To complete the branch migration in the recognition step, 42 consecutive TMD simulations were applied, while the DS RNA release process in the therapeutic step was performed by 37 consecutive TMD simulations. Each TMD simulation was conducted by 3000 steps with 2 fs timestep. The force constant in the TMD simulations was adjusted between 2.0 and 20.0 kcal molÅ^−1^. Both energy minimization and TMD simulations were performed by Amber simulation package.^[94]^

### Cellular Uptake of recNANPs:

PANC-1 (ATCC CRL-1469) and HEK-293FT (ATCC CRL-1573) cells were maintained in Dulbecco’s modified Eagle’s medium, supplemented with 10% heat-inactivated FBS and penicillin/streptomycin (100 U ml^−1^–100 μg ml^−1^). Fluorescent microscopy was used to visualize the uptake of the Alexa-488 labeled recNANPs (strand 1 labeled) into PANC-1 and HEK-293FT. Each of the cell lines was seeded at a density of 40000 cells per well in a 24-well plate containing 200 μL of media and was further maintained for 24 h at 37 °C with 5% CO_2_ in a humidified incubator. Sample preparation of the recNANPs and DS RNAs with Lipofectamine 2000 (L2K) included a 30-min incubation time at room temperature before transfection. After the incubation periods, sample volumes were brought up to 50 μL and were then transfected into the cells. The cells were treated with DS RNAs and recNANPs with L2K and incubated for 48 h at 37 °C with 5% CO_2_. Afterward, the cells were washed with phosphate-buffered saline (1X PBS, pH 7.4) and visualized to assess the presence of the Alexa-488 fluorophore-labeled recNANPs or DS RNAs using the LEICA DMi8 system and a GFP Light Cube.

### Specific Gene Silencing:

PANC-1 and HEK-293FT cells were cultured in 24-well plates at a seeding density of 40000 cells per well and maintained for 24 h at 37° C and 5% CO_2_. Sample preparation of the recNANPs and DS RNAs with DOTAP was done following the manufacturer’s guidelines and using a 1:10 w/w ratio and a 30 min incubation period at room temperature, while sample preparation with L2K followed the listed protocol above. After 24 h, the cells were transfected with either DS RNAs or recNANPs at desired concentrations using either L2K or DOTAP as the transfection agent. Cells were left to incubate in a humidified incubator for 72 h at 37 °C and 5% CO_2_, with media changed following the first 24 h after treatments were added. Once the incubation period ended, the cells were washed using 1X PBS and detached using 0.25% trypsin-EDTA. The cells were spun down at 500 g for 5 min, washed once with PBS, and lysed by using Triton lysis buffer (50 mM Tris-pH 8.0, 150 mM NaCl, and 1% TX-100). The cell lysates were then tested for protein concentration using a BCA assay and evaluated for expression of Survivin by western blot analysis. Samples were electrophoresed and transferred onto membranes. The membranes were blocked with 5% milk for 1 h and then incubated overnight at 4 °C with primary antibodies for Survivin (Abcam, Cat # EP2880Y, kDa of 16.5) or BCL-2 (Cells signaling, Cat# 3498S) or Kras (Cells signaling, Cat# 33197S) and the housekeeping gene, Glyceraldehyde-2-phosphate dehydrogenase (GAPDH) (Santa Cruz, Cat # sc-47724, kDa of 37). The blots were then washed and incubated in the presence of HRP-conjugated secondary anti-rabbit (Abcam, Cat# ab205718) and anti-mouse antibodies (Cells Signaling, Cat# 7076S), respectively. Bound enzymes were detected using a Western Pico ECL kit and the Bio-Rad Chemi-Doc imaging system. Western blots were quantified using the ImageLab software (Bio-Rad).

### Combinatorial Treatment:

PANC-1 cells were plated in a 96-well plate at a seeding density of 5000 cells per well and were maintained for 24 h at 37 °C and 5% CO_2_. recNANPs or DS RNAs were incubated with Lipofectamine 2000 (L2K) for 30 min at room temperature, followed by the addition of each chemotherapeutic (Gemcitabine, Doxorubicin, or Cisplatin), which was prepared as a stock solution of 1 mM, 10 μM, and 1 μM in endotoxin-free water. DMEM (10% (v/v) heat-inactivated fetal bovine serum (FBS), 1% Pen-Step) was added to each treatment to bring the final concentration to 10 and 25 nM for recNANPs and DS RNAs with or without chemotherapeutic from 0.01 to 10 μM per well. Once the experimental time point was reached, CellTiter 96 AQ_ueous_ one-solution cell proliferation assay (MTS, Promega) reagent was added according to the manufacturer’s guidelines. Cell viability was normalized to the cells-only treatment, and a one-way ANOVA statistical test was ran using GraphPad Prism version 9.0.0 for Windows, GraphPad Software, San Diego, California, USA, www.graphpad.com. Data were expressed as the mean ± SEM for a minimum of *n* = 3 independent experimental replicates.

Immunoblotting was performed using DS RNAs or recNANPs with or without chemotherapeutics at 0.1 μM for 72 h to further investigate the effect on protein expression. Protocols for plating and protein extraction were similar to the protocol listed above.

### Peripheral Blood Mononuclear Cells Experiment:

Human donor blood was collected under the IRB-approved NCI-Frederick protocol OH99CN046 D, which specifies details for collecting donor blood for research purposes, including obtaining informed consent from donors prior to blood collection. Human PBMCs were isolated from three different donors, following previously published protocols.^[71,72,77,78]^ PBMCs were then transfected with either re plus lipofectamine 2000 or recNANPs plus DOTAP for 24 h following the earlier protocols. After the 24-h treatment period, the supernatants were collected and used freshly on a multiplex assay.

### Reporter Cell Line and Cell Viability Assays:

HEK-Blue hTLR3, HEK-Blue hTLR7, HEK-Blue hTLR9, and HEK-Lucia RIG-I cell lines were obtained from Invivogen (location). The cells were maintained at 37 °C with 5% CO_2_ and cultured in 96-well flat-bottom Greiner plates according to the manufacturer-specified protocol. Briefly, hTLR3, hTLR7, and RIG-I cells were seeded at ≈50 000 cells per well, while hTLR9 cells were seeded at ≈80 000 cells per well. Positive controls for each cell line included Poly I:C (2 μg mL^−1^) for hTLR3 and hTLR9, R848 (5 μg mL^−1^) for hTLR7, and RNA cube (10 nM) for RIG-I. Post-transfection, cells with their respective treatments were incubated at 37 °C with 5% CO_2_ for 24 h prior to SEAP, IRF activation, and viability testing. For HEK-Blue cells, a QUANTI-Blue assay was conducted in accordance with the protocols to assess the SEAP activation of the treatments. For HEK-Lucia cells, a QUANTI-Luc assay was performed to evaluate IRF activation. Assay results were analyzed using a Tecan Spark plate reader at an absorbance of 638 nm. All samples were normalized to cells alone to assess the treatments of three biological (n = 3) repeats in triplicate. THP1-Dual IRF immune activation and cell viability assays were conducted in accordance with InvivoGen’s protocols, with cells maintained at 37 °C with 5% CO_2_. Seeding was performed using a 96-well flat-bottom Greiner plate, with ≈100 000 cells seeded per well following the manufacturer’s guidelines. Transfections were carried out on the same day using either DOTAP or L2K as carriers for recNANPs. The IRF positive control, 2′3’ cGAMP (2 μg mL^−1^), was included. Following transfection, cells were incubated at 37 °C with 5% CO2 for 24 h before assessing IRF activation and cell viability. IRF activation was evaluated using a QUANTI-Luc assay, while cell viability was determined using an MTS colorimetric assay following the manufacturer’s guidelines. Cell viability results were analyzed using a Tecan Spark plate reader. Samples were normalized to cells alone, and each treatment was performed in triplicate with three biological repeats.^[73,95,96]^

### Spheroids Formation and Uptake:

HEK-293FT and PANC-1 cells were cultured in Dulbecco’s Modified Eagle Medium (DMEM) supplemented with 10% heat-inactivated fetal bovine serum (FBS) and 1% Pen/Strep. Cells were maintained in a humidified atmosphere at 37 °C and 5% CO_2_. Spheroids were generated by seeding 10 000 cells per well, in 200 μl media, into 96-well plates pre-coated with a 1% agarose layer (60 μL per well). Plates were centrifuged at 1,500 rpm for 5 min and subsequently incubated for three days, allowing the formation of compact and uniform spheroids. Three-days-old spheroids were transfected with 10 or 25 nM Alexa-488-labeled DS RNAs or recNANPs, previously complexed with Lipofectamine 2000 for 30 min at room temperature. After 24 h, spheroids were imaged using a Leica DMi8 fluorescent microscope before and after washing and nuclei staining with NucBlue. For NucBlue staining, 7ul of the dye solution was added per well, and the plate was incubated for 20 min protected from light. Spheroids were imaged again at 48 h post-transfection.

### Statistical Analysis:

Experimental results were normalized to untreated controls and were presented as mean ± SEM. The number of biological replicates was indicated in the respective figure legends. Statistical significance was assessed using GraphPad Prism software, with a P-value of < 0.05 considered statistically significant.

## Supplementary Material

SI

Movie 1

Movie 2

Supporting Information is available from the Wiley Online Library or from the author.

## Data Availability

The data that support the findings of this study are available on request from the corresponding author. The data are not publicly available due to privacy or ethical restrictions.
