# Supplementary material for: Reconfigurable Nucleic Acid Nanoparticles with Therapeutic RNAi Responses to Intracellular Disease Markers
Source: Adv Funct Mater. Author manuscript; Available in PMC 2025 Aug 22. (PMC12369976; doi:10.1002/adfm.202508122)
Supplement: SI [file NIHMS2101912-supplement-SI.docx]

Supporting Information for:

**Reconfigurable nucleic acid nanoparticles with therapeutic RNAi responses to intracellular disease markers**

Yelixza I. Avila^1#^, Anh Ha^1#^, Morgan R. Chandler^1,2^, Nathalia Leal Santos^1,3^, Taejin Kim^4^, Hannah S. Newton^5^, Marina A. Dobrovolskaia^5^, Kirill A. Afonin^1^*

^1^ - Nanoscale Science Program, Department of Chemistry, University of North Carolina at Charlotte, Charlotte, NC 28223, USA

^2^ - MIMETAS US, Inc, Gaithersburg, Maryland 20878, USA.

^3^ - Center for Translational Research in Oncology (LIM24), Instituto do Câncer do Estado de São Paulo, Hospital das Clínicas da Faculdade de Medicina da Universidade de São Paulo, Comprehensive Center for Precision Oncology, Universidade de São Paulo, São Paulo 01246-000, Brazil.

^4^ - West Virginia University Institute of Technology, Beckley, West Virginia 25801, USA

^5^ - Nanotechnology Characterization Laboratory, Cancer Research Technology Program, Frederick National Laboratory for Cancer Research, Frederick, MD 21702, USA

#-these authors contributed equally to this project

*- correspondence to Kirill A. Afonin at kafonin@charlotte.edu

**Sequences Used in This Project:**

**recNANP activated by mutated KRAS and targeting Survivin**

*Strand 1:* 5’pGGACCACCGCAUCUCUACAUUCAAG

*Strand 2:* 5’tatcgtcaaggcactcttgCCTACGCCATCAGCTCCGTAGGCTTGAATGTAGAGATGCGGTGGTCC

*Strand 3:* 5’caagagtgccttAAGGACCACCGCATCTCTACATTCAAGcctac

*Strand 4:* 5’CUUGAAUGUAGAGAUGCGGUGGUCCUU

**DS RNA against Survivin**

*Sense (Strand 1):* 5’pGGACCACCGCAUCUCUACAUUCAAG

*Antisense (Strand 4):* 5’CUUGAAUGUAGAGAUGCGGUGGUCCUU

*Alexa488-labeled sense (Al488 Strand 1):* 5’GGACCACCGCAUCUCUACAUUCAAG/3AlexF488N/

**recNANP activated by mutated KRAS and targeting BCL2**

*Strand 1:* 5’pGUACAUCCAUUAUAAGCUGUCGCAG

*Strand 2:* 5’TATCGTCAAGGCACTCTTGCCTACGCCATCAGCTCCGTAGGTTCTGCGACAGCTTATAATGGATGTAC

*Strand 3:* 5’CAAGAGTGCCTTAAGTACATCCATTATAAGCTGTCGCAGCCTAC

*Strand 4:* 5’CUGCGACAGCUUAUAAUGGAUGUACUU

**DS RNA against BCL2**

*Sense (Strand 1):* 5’pGUACAUCCAUUAUAAGCUGUCGCAG

*Antisense (Strand 4):* 5’CUGCGACAGCUUAUAAUGGAUGUACUU

**recNANP-WT activated by WT KRAS and targeting Survivin**

*Strand 1:*  5’pGGACCACCGCAUCUCUACAUUCAAG

*Strand 2-WT:* 5' TATCGTCAAGGCACTCTTGCCTACGCCACCAGCTCCGTAGGCTTGAATGTAGAGATGCGGTGGTCC

*Strand 3:* 5' caagagtgccttAAGGACCACCGCATCTCTACATTCAAGcctac

*Strand 4:* 5’CUUGAAUGUAGAGAUGCGGUGGUCCUU

**Target**:

5’GTAGTTGGAGCTGATGGCGTAGGCAAGAGTGCCTTGACGATACAGCTAATTCAG

**Mock 1:**

5’GGCAACTTTGATCCCTCGGTTTAGCGCCGGCCTTTTCTCCCACACTTTCACG

**Mock 2:**

5’GGGAAUCCAAGGAGGCAGGAUUCCsCGUCACAGAAGGAGGCACUGUGAC

**Scramble recNANP with DS RNA against Survivin**

*Strand 1:* 5’pGGACCACCGCAUCUCUACAUUCAAG

*Strand 2-scramble:* 5'CCGGAACTCATGGCTACTTTTTTTTTTTTGTAGCTTCTTGAATGTAGAGATGCGGTGGTCC

*Strand 3:* 5'CATAGAGTTCCGGAAGGACCACCGCATCTCTACATTCAAGGCTAC

*Strand 4:* 5’CUUGAAUGUAGAGAUGCGGUGGUCCUU

**
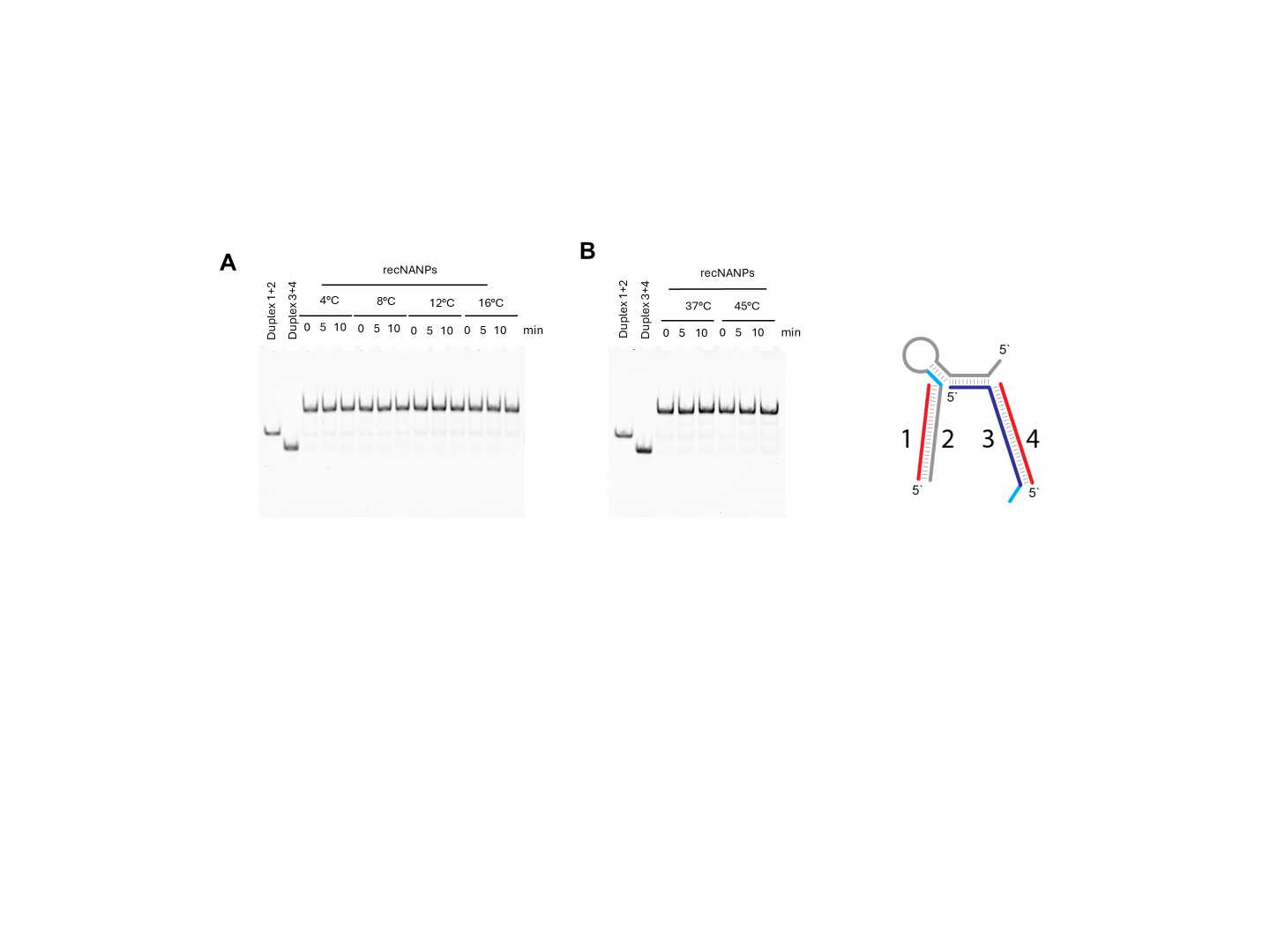
**

**Fig. S1:** Optimization of recNANP assembly conditions. Pre-formed duplexes 1+2 and 3+4 were combined and incubated under varying temperature conditions: (**A**) at 4, 8, 12, and 16 °C, and (**B**) at 37 and 45 °C for 0 to 10 minutes. Assembly products were visualized by native-PAGE using ethidium bromide staining.


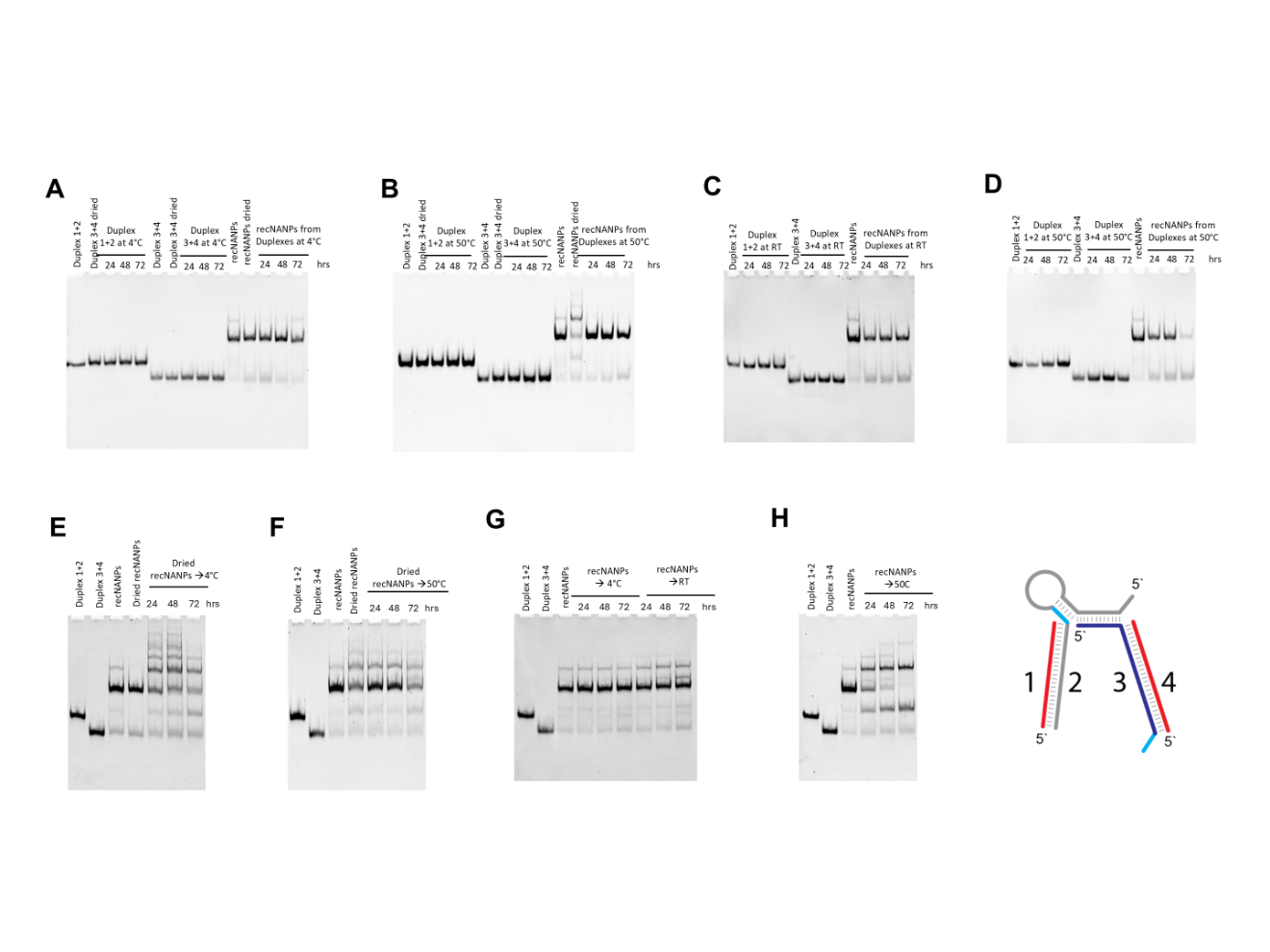


**Fig. S2:** Relative stability of recNANPs assessed after their storage at 4°C, room temperature (RT), and 50°C for durations ranging from 24 to 72 hours. (**A** and **B**) The stability of speed-vac-dried recNANPs and duplexes were assessed when stored at 4°C and 50°C, with the recNANPs assembled using dried duplexes stored from 24 to 72 hours. (**C** and **D**) Duplexes in solution were examined when stored at RT and 50°C, with recNANPs assembled using these stored duplexes from each time points. (**E** and **F**) Dried assembled recNANPs were evaluated when stored at 4°C and 50°C from 24 to 72 hours, as well as the stability of recNANPs in solution stored at 4°C, RT, and 50°C at corresponding time points (**G** and **H**).

**
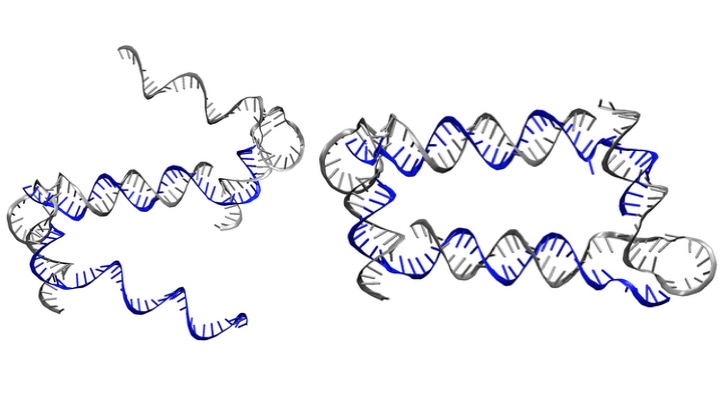
**

**Fig. S3:** Predicted DNA structures resulting from RNase H treatment of recNANPs. In the absence of target strands, RNase H selectively digests RNA within the recNANPs, as these strands are present within RNA/DNA hybrid regions. Strands 2 and 3 each contain 25 nucleotides capable of base pairing. Using these sequences, we modeled two DNA-only structures that could form from the digestion byproducts. These structures were energy-minimized and are predicted to be larger than the original recNANPs. Consequently, they are expected to migrate as higher molecular weight bands above the original recNANP bands in native-PAGE analysis.


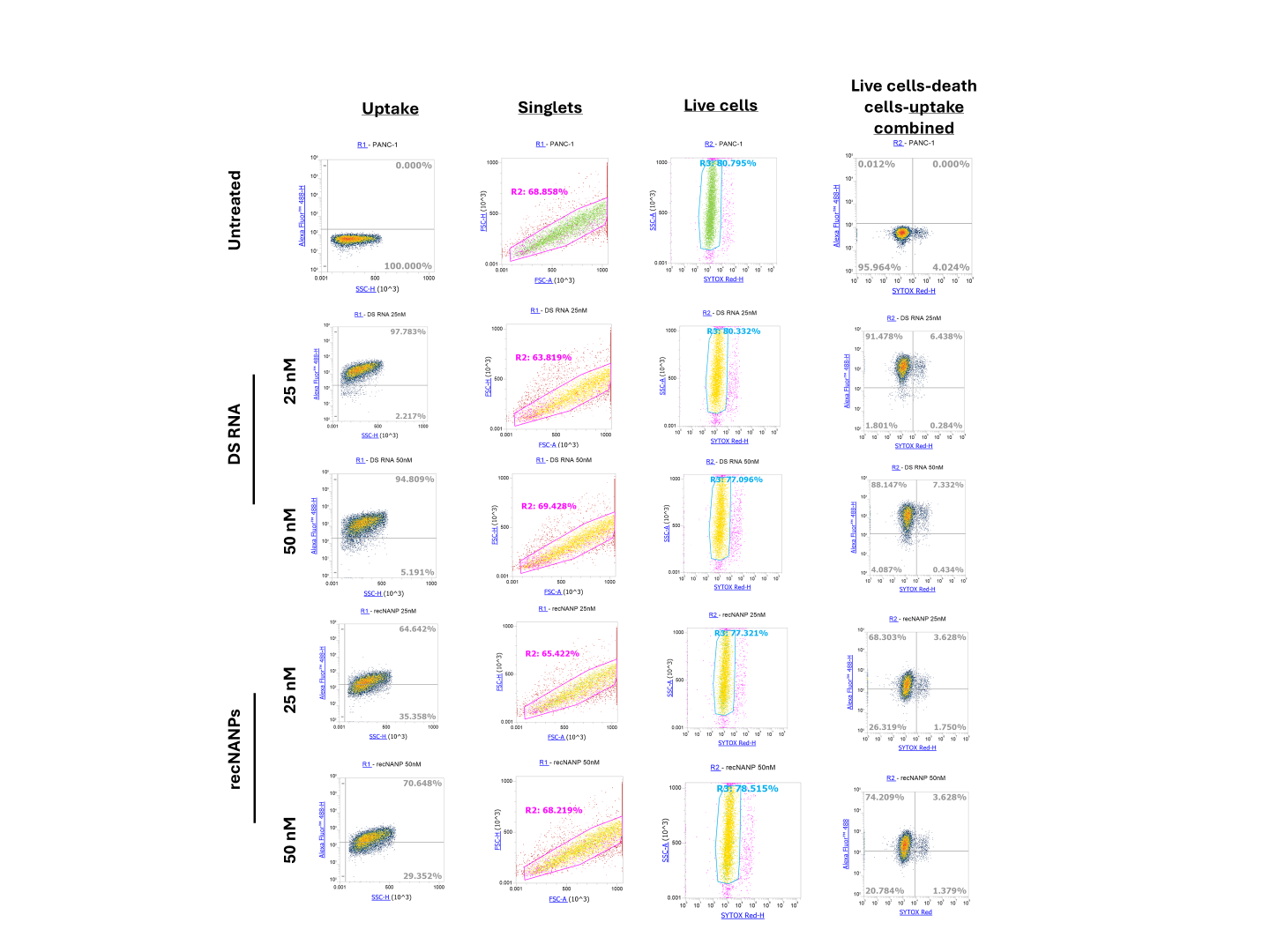


**Fig. S4:** Analysis of PANC-1 cells treated with Alexa 488-labled DS RNAs and Alexa 488-labeled recNANPs. Flow cytometry gating strategies to determine uptake and cell viability for each treatment. Fluorescently labeled DS RNA and recNANPs were transfected at 25 and 50 nM for 48hours.


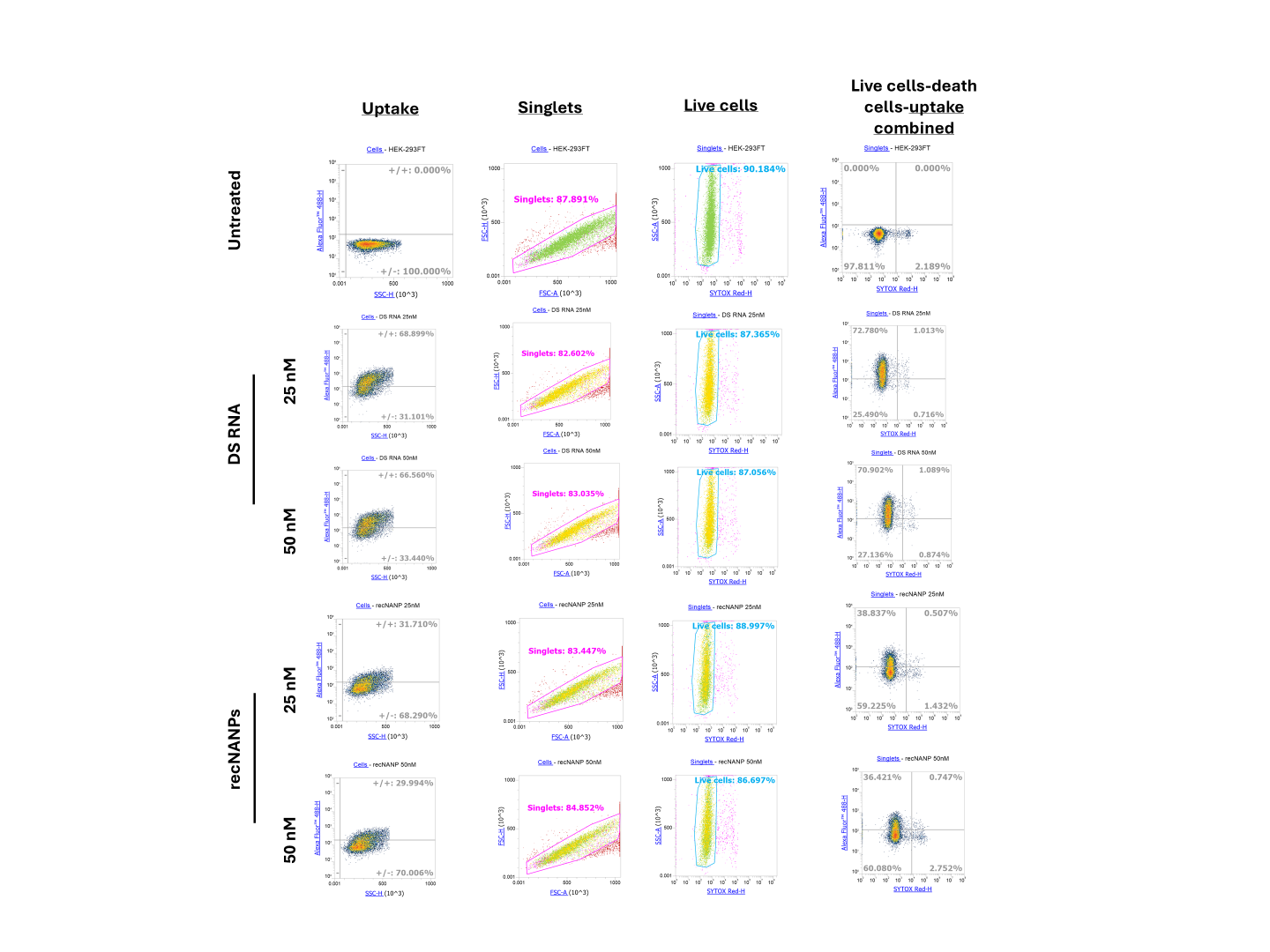


**Fig. S5:** Analysis of HEK-293FT cells treated with Alexa 488-labeled DS RNAs and Alexa 488-labled recNANPs. Flow cytometry gating strategies to determine uptake and cell viability for each treatment. Fluorescently labeled DS RNAs and recNANPs were transfected at 25 and 50 nM for 48hours

**
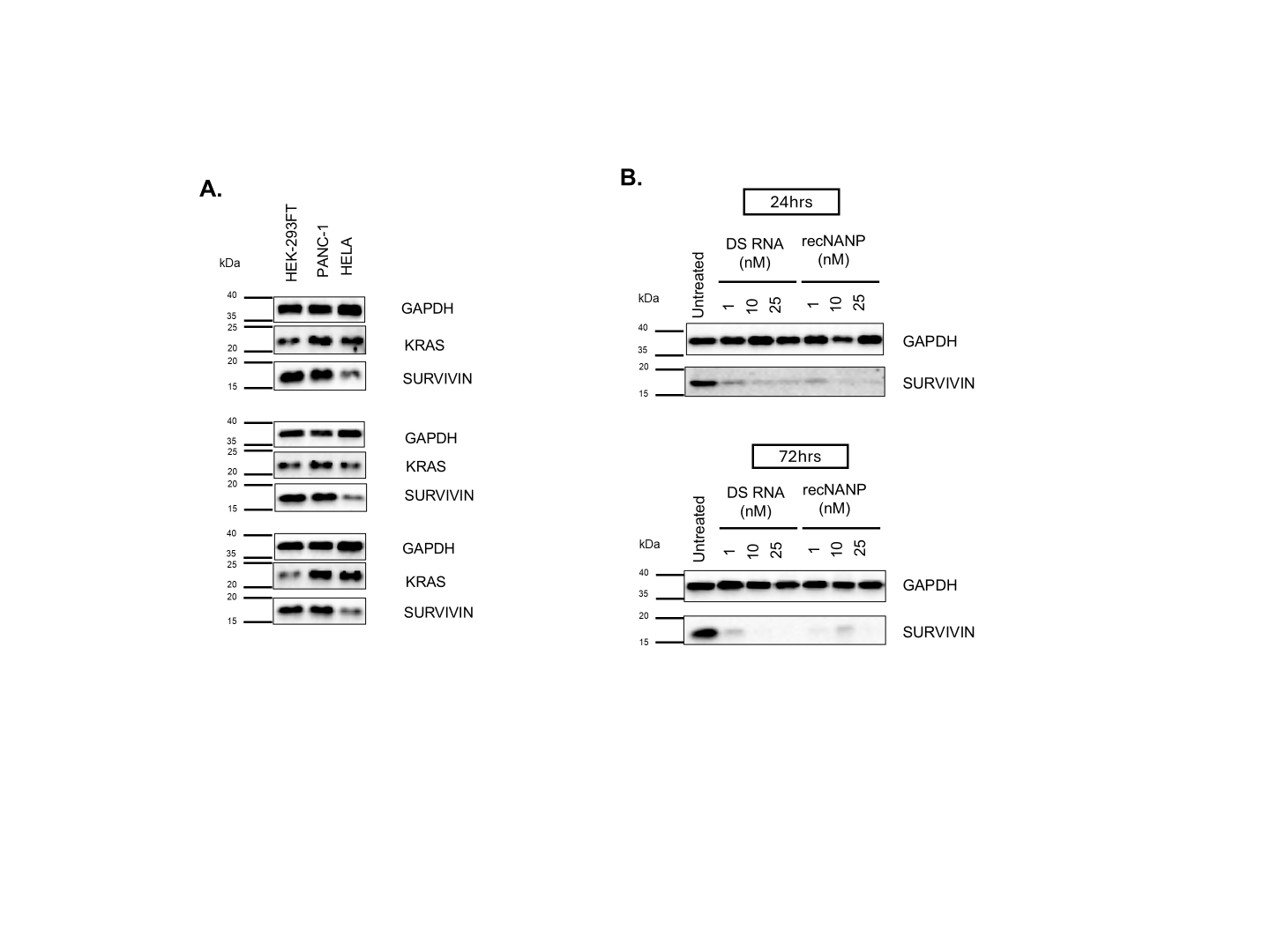
**

**Fig. S6:** (**A**) Relative expression of SURV and KRAS in HEK-293FT, PANC-1, and HeLa cells. **(B)** Immunoblotting of HeLa cells treated with DS RNAs or recNANPs at 1-10-25nM for 24 and 72 hours for the effect on proteins expression.

**
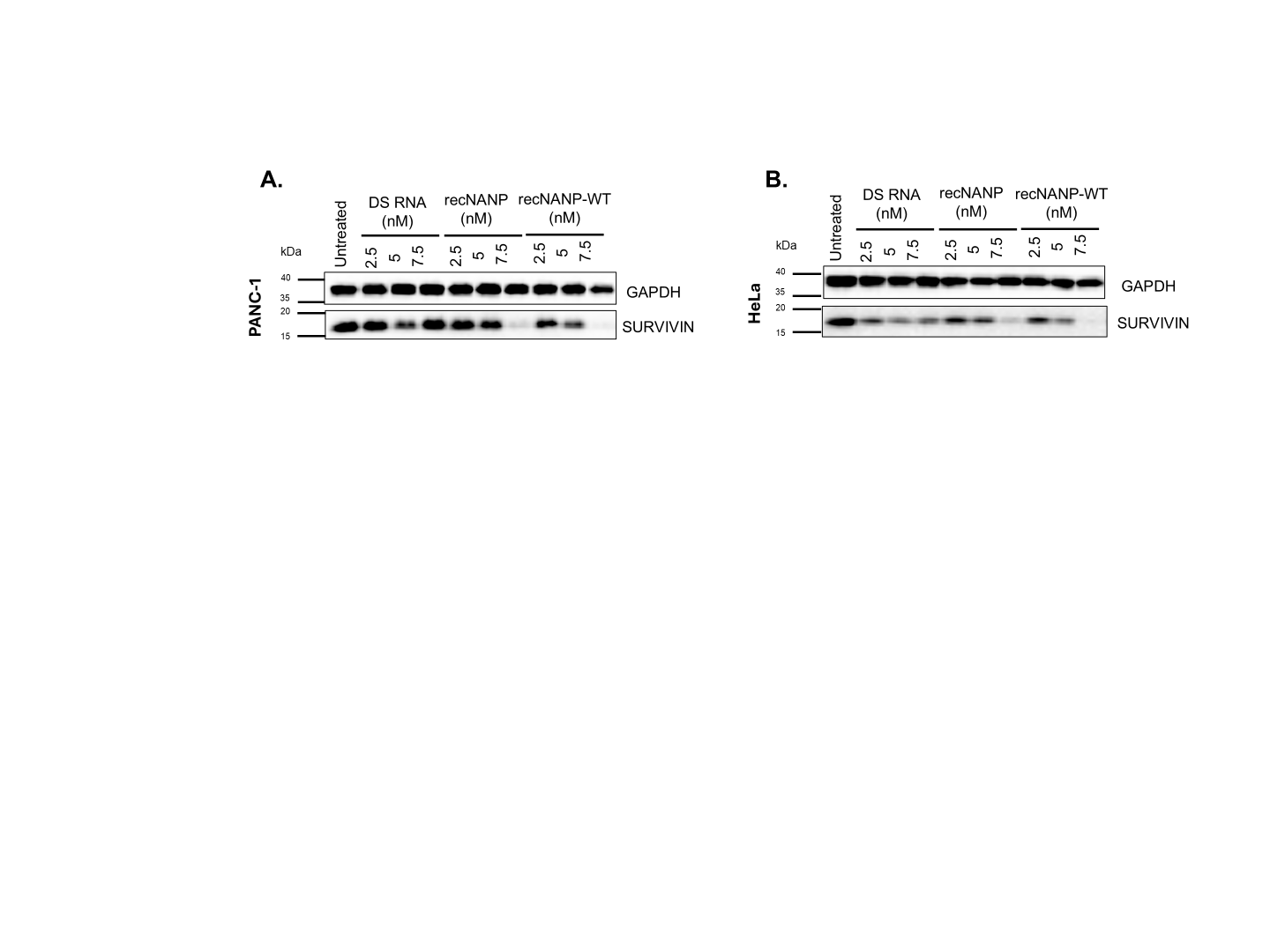
**

**Fig. S7:** Effect of recNANPs targeting mutant KRAS compared to recNANP-WT targeting wild-type KRAS on Survivin expression in **(A)** PANC-1 and **(B)** HeLa at 72 hours.


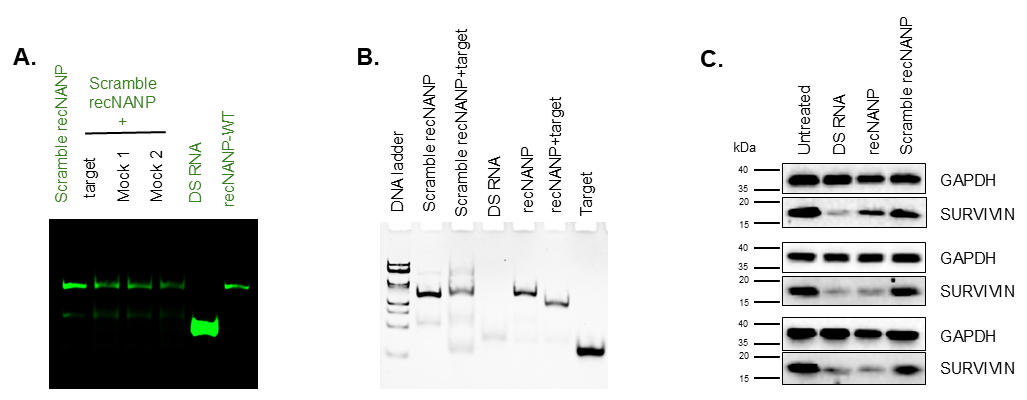


**Fig. S8:** Effects of a scramble stem-loop sequence on recNANP activation, DS RNA release, and Survivin downregulation. (**A**) Alexa 488-labeled scramble recNANPs were incubated with either target strands or mock sequences. No activation and DS RNA release were observed. (**B**) EtBr total staining of scramble recNANPs incubated with target strands show no activation and no DS RNA release. (**C**) Western blot analysis of PANC-1 cells treated with DS RNAs, recNANPs and recNANPs with scrambled stem-loop regions (all at 25 nM, 24 hrs) to assess Survivin protein expression.


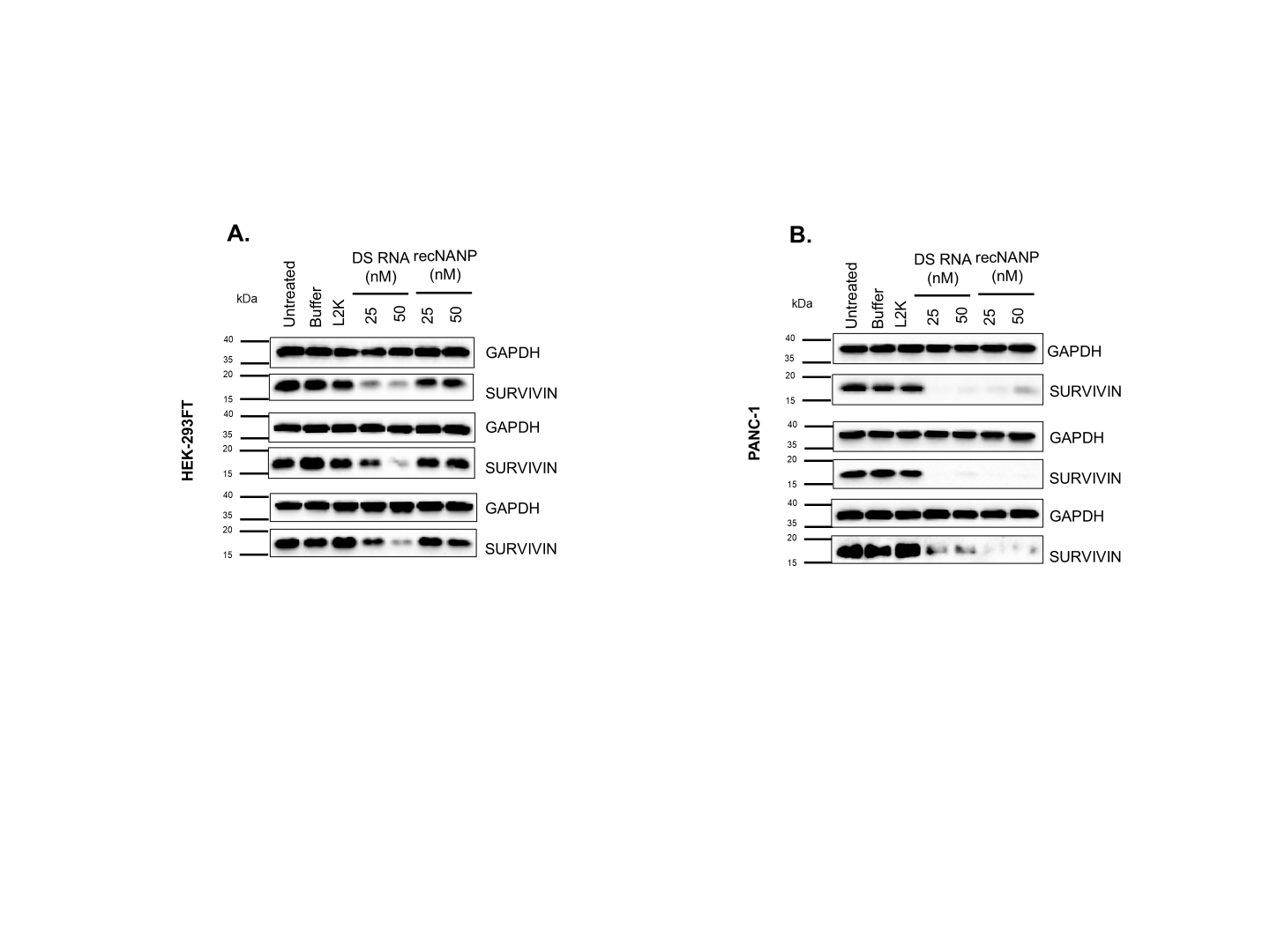


**Fig. S9:** Effect of recNANPs and DS RNAs on Survivin expression in (**A**) HEK-293FT and (**B**) PANC-1 cells.

**
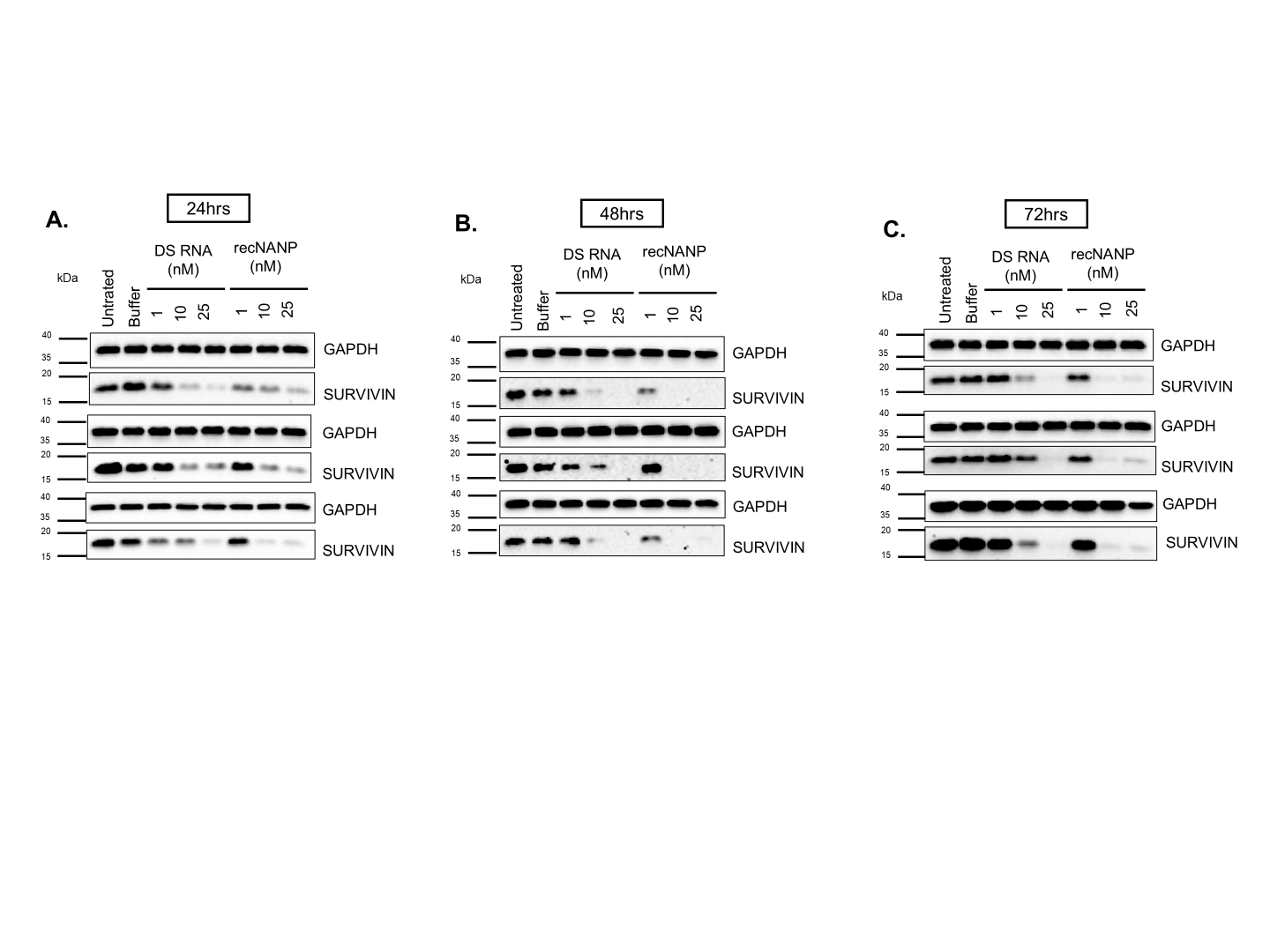
**

**Fig. S10:** Silencing efficiency and duration of Survivin knockdown by recNANPs compared to DS RNAs in PANC-1 cells. Western blots from three biological replicates are shown for each time point: (**A**) 24 hours, (**B**) 48 hours, and (**C**) 72 hours.


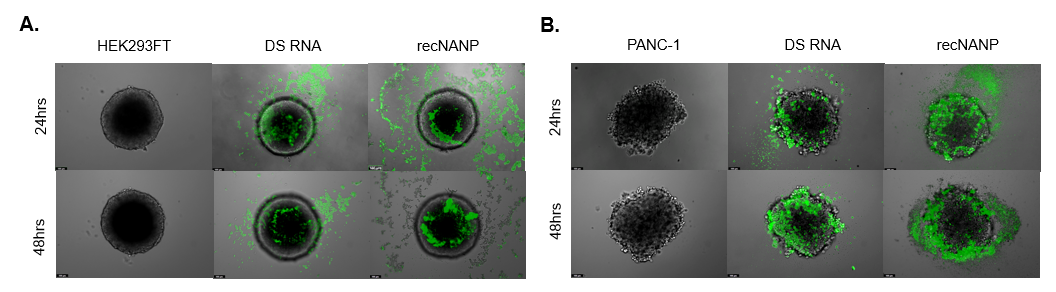


**Fig. S11:** Effect of fluorescently labeled DS RNAs and recNANPs on 3D spheroids at 24- and 48-hours post-transfection, prior to PBS washing. **(A)** HEK-293FT and **(B)** PANC-1


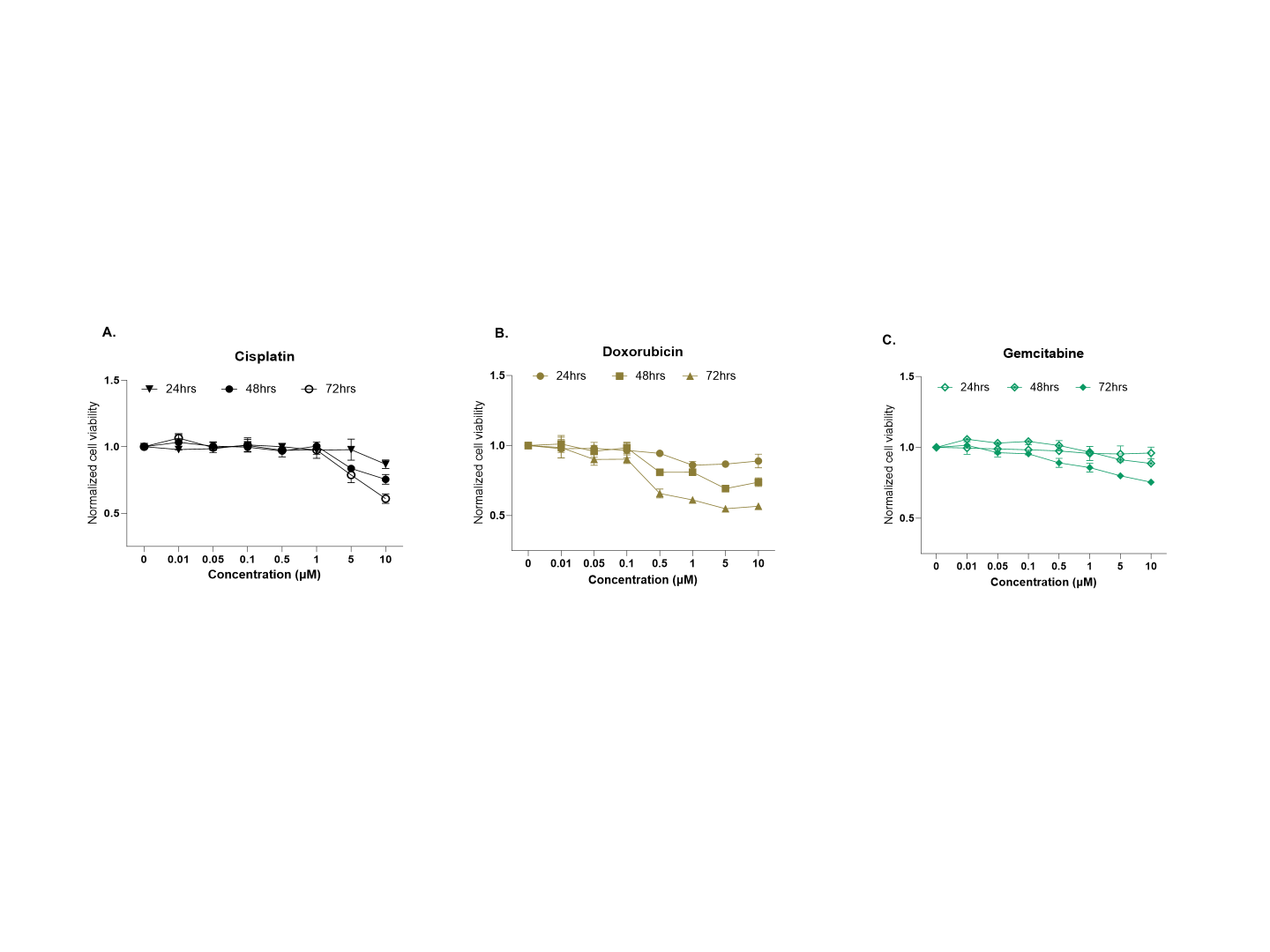


**Fig. S12:** Cell viability assessed in PANC-1 cells treated with Cisplatin, Doxorubicin, or Gemcitabine at concentrations ranging from 0 to 10 µM for 24, 48, and 72 hours. Viability was measured over time in response to each chemotherapeutic agent at increasing concentrations. Data are presented as mean ± SEM (N = 3).

**
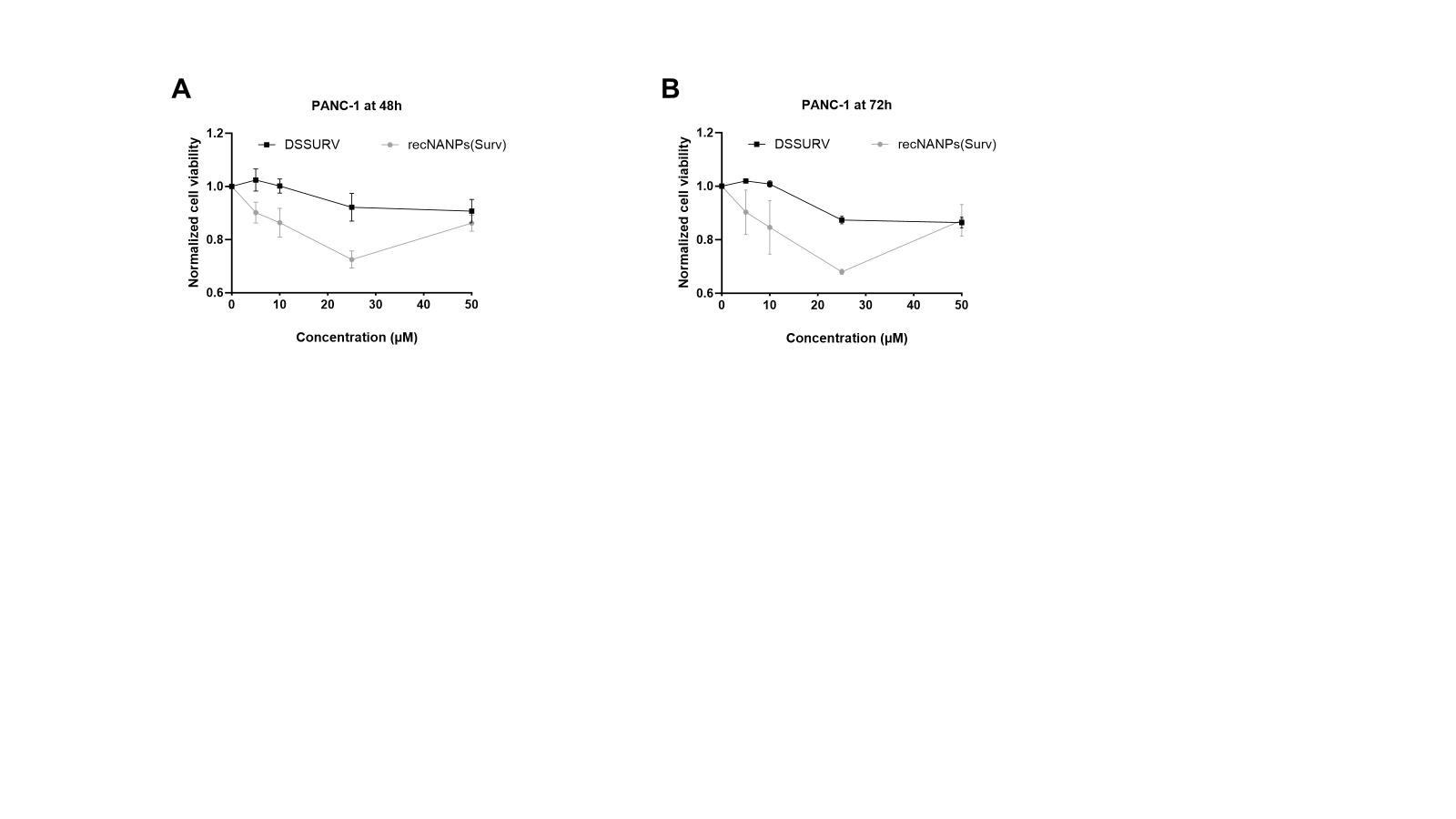
**

**Fig. S13:** PANC-1 cell viability was assessed following treatment with Survivin-targeting DS RNAs or recNANPs at concentrations ranging from 0 to 50 nM for (A) 48 and (B) 72 hours. Data are presented as mean ± SEM (N = 3).


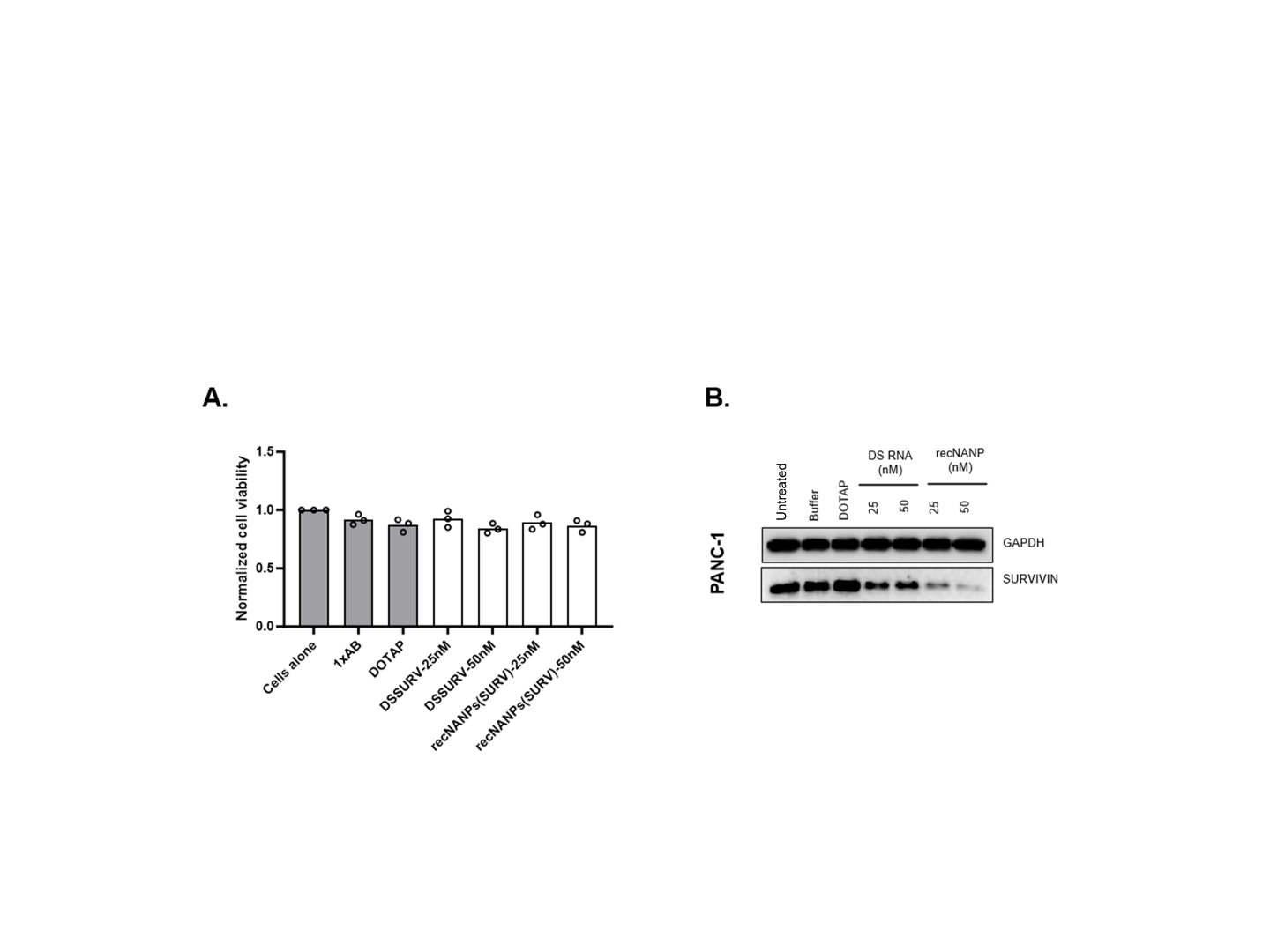


**Fig. S14**: Detection of Survivin expression in PANC-1 cells and cell viability under different concentrations of DS RNAs and recNANPs after 48 hours incubation using DOTAP as a carrier. (A) Cells viability under same treatments in each cell line. Data presented as Mean ± SEM (N=3). (B) Immunoblotting for detection of Survivin in PANC-1 at 25 and 50 nM.

**Fig. S15:** Normalized cell viability after 24 hours of transfection with lipofectamine 2000 (L2K) or DOTAP. **(A)** hTLR 3, hTLR 7, and hTLR 9 viabilities, **(B)** RIG-I viability, **(C)** THP-1 Dual cell viability. Data presented as Mean ± SEM (N=3).


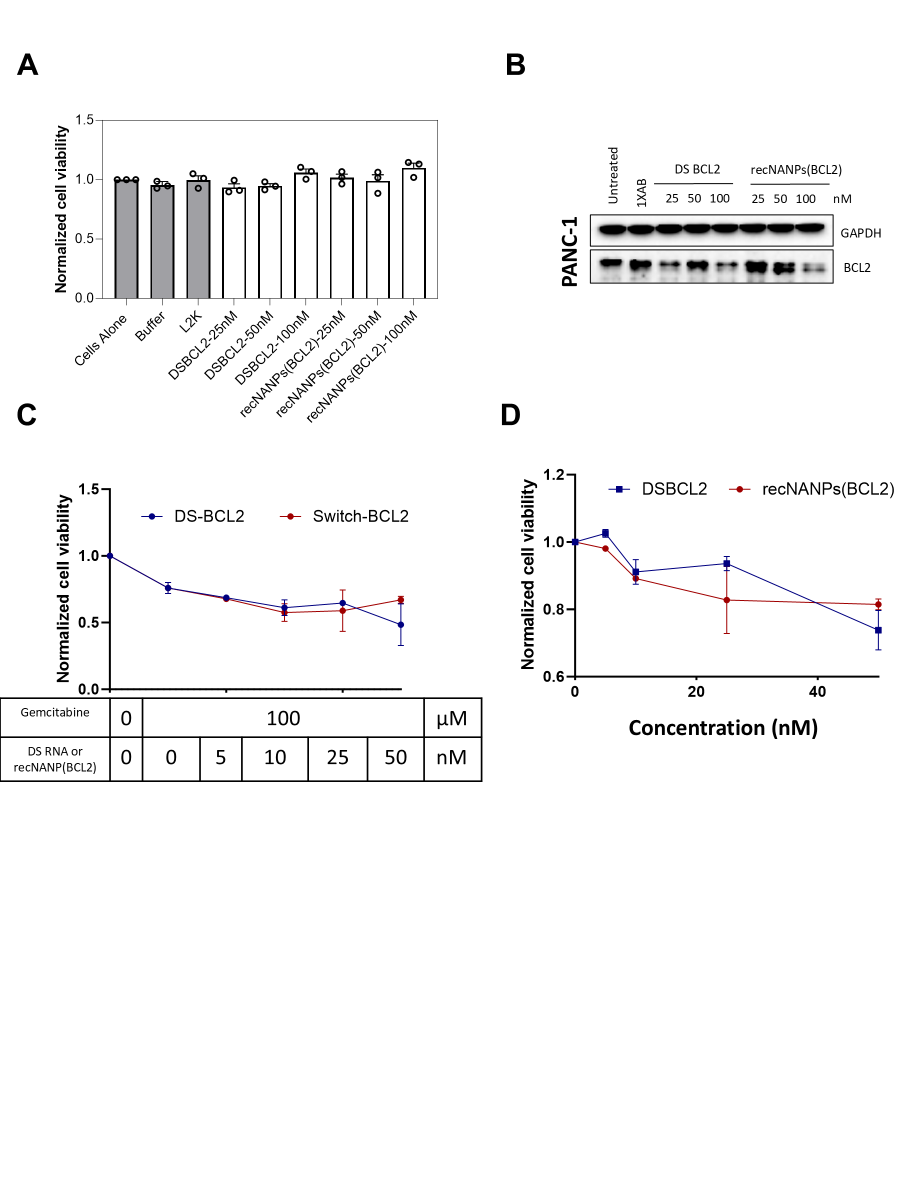


**Fig. S16:** recNANPs (BCL2) effect on BCL2 protein expression in PANC-1. (**A**) Effect of recNANPs (BCL2) on cells viability of PANC-1 after 72 hours of incubation. (**B**) BCL2 regulation in PANC-1 after 72 hours of incubation. (**C**) Combination of Gemcitabine with DS RNA (BCL2) or recNANPs (BCL2) on PANC-1 cells viability in 72 hours of incubation. (**D**) Effect of DS RNA (BCL2) or recNANPs (BCL2) on PANC-1 cells viability in 72 hours of incubation.
